# Rates and risk factors for major adverse cardiovascular and cerebrovascular events after stroke due to intracerebral hemorrhage: Systematic review and study-level meta-analysis

**DOI:** 10.1177/17474930261416692

**Published:** 2026-01-28

**Authors:** Vega Pratiwi Putri, Neshika Samarasekera, Tom J Moullaali, Saketh Jampana, Joseph Aked, Signild Åsberg, Sam Schulman, Georgios Tsivgoulis, Maria Pikilidou, Hsin-Hsi Tsai, Li-Kai Tsai, Phyo Kyaw Myint, Tiberiu A Pana, Charlotte Cordonnier, Barbara Casolla, David Gaist, Alessandro Pezzini, Pol Camps-Renom, Catharina J.M. Klijn, Michele Romoli, Arnstein Tveiten, Ming Liu, Mangmang Xu, Bo Wu, David Werring, Philip S Nash, Gargi Banerjee, Linxin Li, Rustam Al-Shahi Salman

**Affiliations:** 1 Institute for Neuroscience and Cardiovascular Research, The University of Edinburgh, Edinburgh, UK; 2 Department of Neurology, Universitas Gadjah Mada, Yogyakarta, Indonesia; 3 Edinburgh Medical School, The University of Edinburgh, Edinburgh, UK; 4 Department of Clinical Sciences, Neurology, Lund University, Lund, Sweden; 5 Department of Medicine, Blekinge Hospital, Karlskrona, Sweden; 6 Department of Medical Sciences, Uppsala University, Uppsala, Sweden; 7 Department of Medicine, McMaster University and Thrombosis and Atherosclerosis Research Institute, Hamilton, ON, Canada; 8 Second Department of Neurology, National and Kapodistrian University of Athens, School of Medicine, Athens, Greece; 9 Second Department of Nephrology, Hypertension Excellence Center, AHEPA Hospital, Aristotle University of Thessaloniki, Thessaloniki, Greece; 10 Department of Neurology, National Taiwan University Hospital, Taipei; 11 Ageing Clinical and Experimental Research (ACER) Team, Institute of Applied Health Sciences, School of Medicine, Medical Sciences and Nutrition, University of Aberdeen, Aberdeen, UK; 12 University of Lille, Inserm, CHU Lille, U1172, Lille, France; 13 Stroke Unit, CHU Pasteur 2, Université Cote d'Azur, UMR2CA, Nice, France; 14 Research Unit for Neurology, Odense University Hospital, Odense, Denmark; University of Southern Denmark, Odense, Denmark; 15 Department of Medicine and Surgery, University of Parma, Parma, Italy; 16 Stroke Care Program, Department of Emergencies, Parma University Hospital, Parma, Italy; 17 Neurology Department, Hospital de la Santa Creu i Sant Pau, Barcelona, Spain; 18 Department of Neurology, Donders Institute for Brain, Cognition and Behaviour, Radboud University Medical Centre, Nijmegen, Netherlands; 19 Department of Neurology and Neurosurgery, Brain Center, University Medical Center Utrecht, Utrecht, Netherlands; 20 Neurology Unit, Rimini “Infermi” Hospital—AUSL Romagna, Rimini, Italy; 21 Department of Neurology, Hospital of Southern Norway, Kristiansand, Norway; 22 Department of Neurology, Center of Cerebrovascular Diseases, West China Hospital, Sichuan University, Chengdu, China; 23 Stroke Research Centre, Department of Translational Neuroscience and Stroke, UCL Queen Square Institute of Neurology and the National Hospital for Neurology and Neurosurgery, London, UK; 24 UCL Institute of Prion Diseases, MRC Prion Unit at UCL, London, UK; 25 Wolfson Centre for Prevention of Stroke and Dementia, Nuffield Department of Clinical Neurosciences, University of Oxford, Oxford, UK

**Keywords:** Intracerebral hemorrhage, stroke, stroke recurrence, MACE, prognosis, systematic review

## Abstract

**Background:**

Intracerebral hemorrhage (ICH) survivors are at increased risk of major adverse cardiovascular and cerebrovascular events (MACE) compared with population controls; however, little is known about the annual rates and risk factors for MACE.

**Methods:**

We searched Medline, Embase, and trial registries systematically in April 2024 for studies of adults with ICH, reporting either a MACE composite outcome or both ischemic and hemorrhagic outcomes, with at least one year of follow-up. We excluded studies limited to secondary ICH or isolated non-ICH intracranial hemorrhages. We used the QUIPS tool to assess studies’ risk of bias. The primary outcome was the rate of MACE. We used a random-effects meta-analysis to estimate the annual event rate (per 100 person-years, expressed as %) for each outcome. We conducted subgroup analyses and meta-regression to explore heterogeneity.

**Results:**

We included 26 studies, involving 198,289 ICH survivors. Individual studies’ reported annual rate of MACE ranged 4.2–14.6%. The pooled annual rate of recurrent ICH was 2.1% (95% confidence interval (CI) = 1.7–2.6; 26 studies; I^2^ = 94%) and of ischemic stroke was 2.0% (95% CI = 1.5–2.7; 24 studies; I^2^ = 95%). Meta-regression analyses identified one statistically significant association between a higher prevalence of atrial fibrillation and an increased risk of ischemic stroke.

**Discussion:**

The rates of recurrent ICH and ischemic stroke were comparable among ICH survivors, but evidence about other MACE outcomes remains limited. An individual participant data meta-analysis is needed to investigate the predictors of MACE outcomes, which may help inform risk stratification and prognosis among ICH survivors.

## Graphical abstract

**Graphical abstract:**
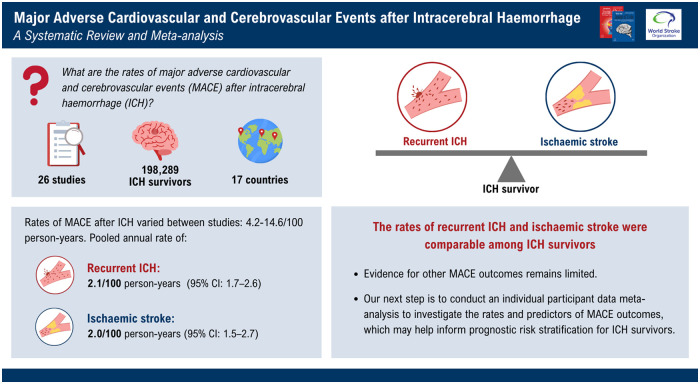

## Introduction

Stroke is a leading cause of death and disability-adjusted life years (DALYs), with intracerebral hemorrhage (ICH) responsible for approximately half of stroke-related DALYs, despite being less common than ischemic stroke.^1^ ICH survivors are at high risk of major adverse cardiovascular and cerebrovascular events (MACE), such as recurrent ICH, ischemic stroke, and myocardial infarction.^2^

More is known about the rate of recurrent ICH than ischemic events after ICH, but there are no systematic reviews with meta-analyses of the rates of these outcomes, despite an increasing number of relevant studies^3–6^ published since the last meta-analysis in 2013.^7^ Furthermore, relatively little is known about the risk factors for MACE and MACE subtypes. To date, atrial fibrillation has been associated with an increased rate of ischemic MACE after ICH,^3,6,8,9^ while lobar hemorrhage^3,6,10^ and cerebral amyloid angiopathy (CAA) imaging biomarkers^9^ have been associated with an increased rate of recurrent ICH. A multivariable analysis from a cohort study reported that male sex, history of coronary artery disease, diabetes, hypercholesterolemia, and nonuse of antithrombotic or statin medications were independently associated with the risk of ischemic MACE, whereas only lobar ICH contributed significantly to the risk of hemorrhagic MACE. This study also developed a prediction model for ischemic stroke, which was internally validated.^11^ However, further research is needed to identify the risk factors for MACE and its specific subtypes and whether these vary by time after ICH, country, or healthcare settings.

Therefore, we aimed to quantify the rates of MACE and its subtypes and identify the risk factors associated with MACE subtypes and subtype components after ICH in an updated systematic review and study-level meta-analysis.

## Methods

We conducted this systematic review and study-level meta-analysis according to a protocol that was written prospectively in accordance with PRISMA-P guidelines^12^ and registered in PROSPERO (CRD42024555070).

### Eligibility criteria

We included cohort studies or randomized controlled trials involving adults aged ⩾ 18 years at the time of diagnosis of first-ever or recurrent spontaneous (nontraumatic) symptomatic ICH after 2001, when the PROGRESS trial demonstrated the benefits of blood pressure lowering after ICH, influencing secondary prevention practices and making data collected 2001 onward more representative of current care.^13,14^ Eligible studies had at least one year of follow-up and reported overall MACE or at least one component of ischemic MACE (e.g. ischemic stroke or myocardial infarction) and one component of hemorrhagic MACE (e.g. recurrent ICH or extracranial hemorrhage). If several publications arose from the same cohort, we included the report with the largest sample size. We excluded studies of ICH due to an underlying macrovascular cause and studies reporting mixed causes of intracranial hemorrhage, where spontaneous ICH could not be separated from others.

### Information sources and search strategy

VP searched Ovid Medline, Embase, and ClinicalTrials.gov on 9 April 2024, for published articles using search strategies that combined terms for ICH,^15^ MACE, and cohort studies without language restrictions (Supplementary Tables 1–3). We excluded conference abstracts. VP imported all records into Covidence, which removed the duplicates.

### Study selection process

Two reviewers (from VP, NS, and SJ) screened each title and abstract independently to determine eligibility. Any uncertainties were resolved through discussion or consultation with a third reviewer (TJM/RA-SS) to reach a decision.

### Data collection process

Two reviewers (from VP, NS, TJM, and RA-SS) independently extracted data from each included study using a standardized proforma. We resolved any disagreements through discussion. If the outcome data were unclear, we contacted the study authors via email for clarification.

### Data items

We recorded study characteristics (mid-year, setting, country, case ascertainment, follow-up methods, and duration of follow-up), participant baseline demographics, and MACE outcomes. We extracted definitions of MACE and its components to identify variability in outcome classification and ensure consistency in data synthesis and interpretation. We extracted the annual incidence rate (number of events per 100 person-years) of MACE, ischemic MACE, hemorrhagic MACE, and key components of MACE subtypes (recurrent ICH, ischemic stroke, and myocardial infarction). For studies not reporting annual rates, we calculated the annual rate by dividing the number of events by the total person-years of follow-up. If a study had multiple follow-up time points, we used the longest follow-up duration for analysis. Furthermore, we extracted data on study characteristics for subgroup analysis and heterogeneity assessment.

### Risk of bias in individual studies

We assessed the risk of bias using the Quality in Prognosis Studies (QUIPS) tool across six domains, with study participants, attrition, and outcome measurement pre-specified as key domains. Two reviewers (from VP, NS, TJM) assessed the risk of bias for each study independently and resolved any discrepancies with a third reviewer. We determined that a study had an overall low risk of bias if it had at least two domains rated as low risk among the three pre-specified key domains and no domains rated as high; otherwise, we classified studies as having moderate or high risk of bias.

### Synthesis methods

The primary outcome was the annual incidence rate (per 100 person-years of follow-up, expressed as % per year) of MACE. Secondary outcomes were the annual rates of each MACE component. We also extracted study-level associations between risk factors for MACE over the entire follow-up period in each study.

We pooled annual rates using a Poisson generalized linear mixed model (GGLM) with random effects (metarate(), *meta* version 8.1-0, R version 4.5.1).^16,17^ We quantified between-study heterogeneity using the I-squared statistic. The results are presented as forest plots.

We pre-specified subgroup analyses by study risk of bias, design (hospital-based vs population-based), region (Asia vs others), inception point (time interval from ICH onset to the beginning of follow-up < 1 month vs > 1 month), and follow-up method (prospective vs retrospective). In a post hoc sensitivity analysis, we assessed variation by the outcome definitions used. We also performed meta-regression to assess the association between study-level characteristics and the rates of recurrent ICH and ischemic stroke.

## Results

Our literature search identified 4051 unique studies to screen. After abstract screening, 3790 were excluded, and a further 224 were excluded after full text review, leaving 37 studies that fulfilled the eligibility criteria (Supplementary Figure 1), but it was not possible to include eight studies for which the authors did not respond to requests for clarification of aggregate data. We excluded three studies^4,18,19^ from which we were unable to calculate the annual rate of outcomes per person-year of follow-up, leaving 26 eligible studies of 198,289 participants from 17 countries: Norway,^10^ Sweden,^20–22^ Denmark,^3^ France,^9^ Germany,^23^ Italy,^11,24^ Greece,^25^ Spain,^26^ Netherlands,^27^ United Kingdom,^6,28,29^ United States of America,^30^ Canada,^20^ Korea,^31^ China,^32–35^ Thailand,^36^ Hong Kong,^30^ and Taiwan.^37,38^

Ten studies had a low risk of bias, 13 moderate, and three were high risk (the latter due to unclear methods of identifying outcomes in two studies^35,38^ and potential selection bias, as participants were limited to those with atrial fibrillation and prior ICH who underwent left atrial appendage occlusion in one study;^26^ Supplementary Figures 2 and 3).

The follow-up duration ranged from 1 to 6 years, and the mid-year of cohort enrollment ranged from 2004 to 2019. Twenty-three studies were cohort studies (of which 20 were hospital-based^3,9–11,20,22–24,26,30–40^ and three were population-based^6,21,25^) and the remaining three studies were randomized controlled trials.^27–29^ Sixteen studies used prospective methods for both case identification and follow-up^6,9,11,23–25,27–29,33–35,37–39,30^; two used retrospective identification with prospective follow-up^22,32^; and eight studies used retrospective methods for both identification and follow-up^3,10,20,21,26,31,36,40^ (Supplementary Table 4).

### Annual rate of MACE, MACE subtypes, and MACE components

Eight studies reported a MACE outcome,^3,6,26–29,31,37^ eight studies reported an ischemic MACE subtype outcome,^3,9,11,24,27,32,40,41^ and five studies reported hemorrhagic MACE subtype outcome^3,9,11,27,41^ (Table 1).

**Table 1.** Annual rate of the major adverse cardiovascular and cerebrovascular event (MACE) primary outcome, MACE subtypes, and MACE components in included studies.

Study	Recurrent ICH		Ischemic stroke		Myocardial Infarction		Hemorrhagic MACE		Ischemic MACE		All MACE	
	N	Rate (95% CI)	N	Rate (95% CI)	N	Rate (95% CI)	N	Rate (95% CI)	N	Rate (95% CI)	N	Rate (95% CI)
Weimar^23^	11	1.13 (0.46–1.8)	21	2.16 (1.24–3.09)	–	–	–	–	–	–	–	–
Majeed^20^	5	4.5 (0.57–8.43)	10	9.0 (3.44–14.56)	–	–	–	–	–	–	–	–
Casolla^9^	24	1.05 (0.63–1.46)	33	1.44 (0.95–1.93)	16	0.7 (0.36–1.04)	31	1.35 (0.88–1.83)	54	2.35 (1.73–2.98)	–	–
Tveiten^10^	4	0.63 (0.01–1.26)	5	0.79 (0.1–1.49)	–	–	–	–	–	–	–	–
Asberg^22^	234	1.2 (1.05–1.35)	350	1.8 (1.61–1.99)	–	–	–	–	–	–	–	–
Gaist^3^	538	1.44 (1.32–1.56)	571	1.52 (1.4–1.65)	194	0.52 (0.45–0.6)	1268	3.61 (3.42–3.81)	1311	3.66 (3.47–3.87)	1547	4.16 (3.96–4.37)
Moon^31^	41	1.22 (0.84–1.59)	26	0.77 (0.47–1.07)	6	0.18 (0.0–0.47)	–	–	–	–	303	8.98 (7.97–9.99)
Pezzini^11^	219	3.0 (2.6–3.4)	108	1.5 (1.22–1.78)	39	0.5 (0.34–0.66)	231	3.2 (2.79–3.61)	169	2.3 (1.95–2.65)	–	–
Ma^32^	39	2.78 (1.91–3.65)	–	–	–	–	–	–	81	5.77 (4.51–7.03)	–	–
Szlachetka^36^	9264	2.61 (2.56–2.66)	4244	1.2 (1.16–1.24)	–	–	–	–	–	–	–	–
Li^6^	15	2.4 (1.3–4.0)	7	1.1 (0.4–2.3)	–	–	–	–	–	–	35	5.7 (4.00–7.90)
Tsivgoulis^25^	0	0.0 (0.0–0.0)	1	1.2 (0.06–9.4)	–	–	–	–	–	–	–	–
Li^6^	31	3.9 (2.7–5.6)	18	2.3 (1.3–3.6)	–	–	–	–	–	–	83	10.6 (8.40–13.30)
Teo^30^	129	4.2 (2.9–8.2)	43	1.4 (0.7–2.1)	40	1.2 (0.7–2.0)	–	–	–	–	–	–
Sembolini^24^	10	3.17 (1.21–5.14)	–	–	–	–	–	–	12	3.81 (1.65–5.96)	–	–
Banerjee^39^	45	1.88 (1.33–2.43)	70	2.93 (2.24–3.61)	–	–	–	–	–	–	–	–
Xu^33^	7	0.98 (0.25–1.7)	6	0.84 (0.17–1.5)	–	–	–	–	–	–	–	–
Teo^30^	37	2.9 (2.0–3.6)	25	1.6 (1.1–2.7)	29	1.8 (1.0–3.0)	–	–	–	–	–	–
Ou^35^	11	3.59 (1.47–5.71)	21	6.86 (3.93–9.79)	–	–	–	–	–	–	–	–
Jung^40^	14	2.03 (0.99–3.07)	8	1.1 (0.34–1.87)	4	0.55 (0.01–1.09)	–	–	15	2.18 (1.11–3.25)	–	–
Xu^34^	8	4.91 (1.51–8.31)	13	7.98 (3.64–12.31)	–	–	–	–	–	–	–	–
RESTART Collaboration^29^	47	2.6 (1.86–3.35)	67	3.71 (2.82–4.6)	21	1.16 (0.67–1.66)	62	3.43 (2.58–4.29)	110	6.09 (4.96–7.23)	159	8.81 (7.44–10.18)
Aked^21^	0	0.0 (0.0–0.0)	1	0.01 (0.0–0.18)	–	–	–	–	–	–	–	–
Tsai^37^	36	6.1 (4.09–8.11)	12	1.9 (0.82–2.98)	–	–	–	–	–	–	47	8.2 (5.85–10.55)
Moliner–Abos^26^	5	2.74 (0.34–5.14)	3	1.64 (0.53–5.09)	–	–	–	–	–	–	16	8.76 (4.47–13.05)
Schreuder^27^	5	2.25 (0.28–4.23)	12	5.41 (2.35–8.46)	2	0.9 (0.1–3.2)	9	4.05 (1.41–6.7)	17	7.66 (4.02–11.3)	26	11.71 (7.21–16.21)
Chen^38^	5	1.27 (0.16–2.39)	10	2.55 (0.97–4.13)	–	–	–	–	–	–	–	–
SoSTART Collaboration^28^	11	4.66 (2.33–8.34)	20	8.96 (5.47–13.84)	2	0.85 (0.0–2.02)	–	–	–	–	31	14.62 (10.0–20.64)

ICH: intracerebral hemorrhage; MACE: major adverse cardiovascular and cerebrovascular events; N: number of outcome events; Rate: outcome events per 100 person-years of follow-up.

Due to the substantial heterogeneity in the definitions of MACE outcomes (Supplementary Table 5–7), we did not perform a pooled meta-analysis. The annual event rates for MACE, ischemic MACE, and hemorrhagic MACE varied across studies. MACE rates ranged from 4.2% to 14.6%, ischemic MACE rates ranged from 2.2% to 7.7%, and hemorrhagic MACE rates ranged from 1.4% to 4.1%.

**Figure 1.**
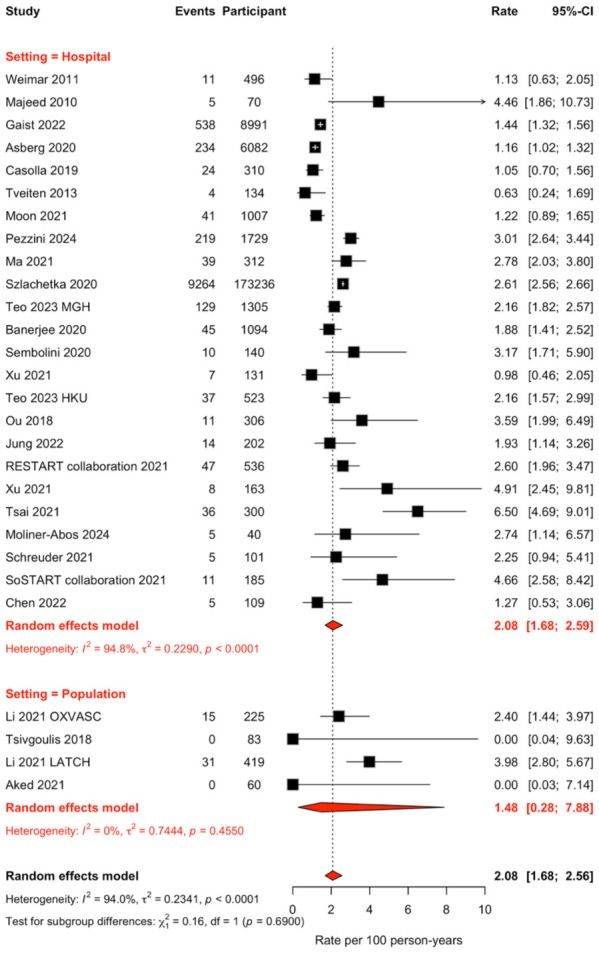
Forest plot of the annual rate of recurrent ICH stratified by study setting. Studies are organized in ascending chronological order of cohort mid-year. Error bars are 95% CIs, and the diamonds represent the pooled estimates.

We separately pooled the annual rates (per 100 person-years of follow-up, expressed as % per year) of recurrent ICH, ischemic stroke, and myocardial infarction, as these were the most frequently reported individual components of MACE (Supplementary Table 8 and Supplementary Figure 4). Among 198,289 participants, with 10,795 recurrent ICH events during 445,983 person-years of follow-up in 26 studies, the pooled annual rate of recurrent ICH was 2.1% (95% CI = 1.7–2.6), with substantial heterogeneity across studies (I^2^ = 94%). We found in subgroup analyses that the annual rate of recurrent ICH remained consistent across study settings (Figure 1) and regions (Supplementary Figure 5), but the rate was higher in studies recruiting participants within 30 days of ICH onset compared with later recruitment (Supplementary Figure 6). This difference may reflect the exclusion of early outcome events by studies of 30-day survivors.

**Figure 2.**
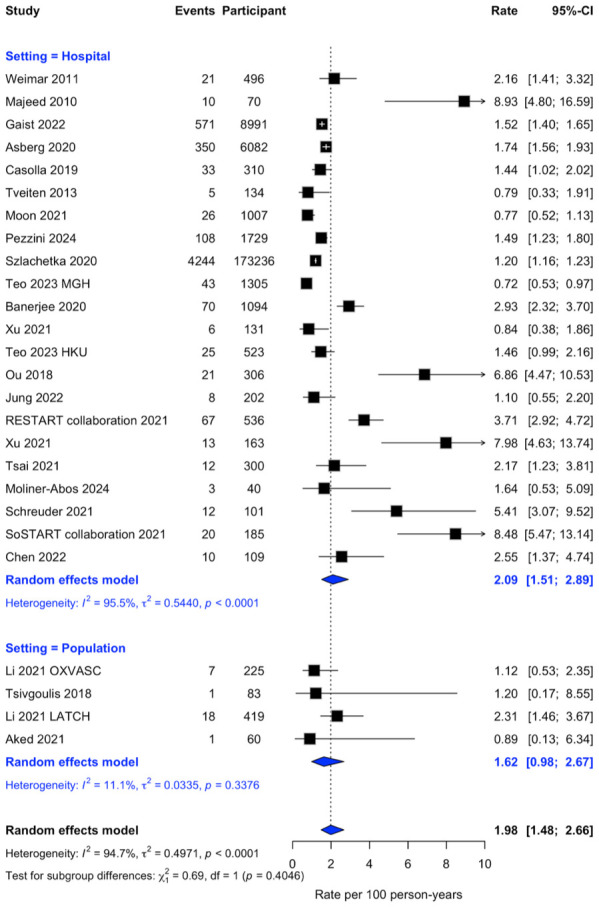
Forest plot of the annual rate of ischemic stroke stratified by setting. Studies are organized in ascending chronological order of cohort mid-year. Error bars are 95% CIs, and the diamonds represent the pooled estimates.

Among 197,797 participants with 5702 ischemic strokes during 444,264 person-years of follow-up in 24 studies, the pooled annual rate of ischemic stroke was 2.0% (95% CI = 1.5–2.7), with substantial heterogeneity across studies (I^2^ = 94.9%). Subgroup analyses showed consistent rates across settings (Figure 2) and regions (Supplementary Figure 7), but the rate was higher in studies with inception within one month of ICH compared with later inception (Supplementary Figure 8). However, the two studies driving this finding included populations with an inherently higher risk of ischemic events, as one study included only ICH survivors with atrial fibrillation,^28^ and the other included anticoagulant-related ICH.^20^ Thus, the higher rates may also reflect the clinical characteristics of specific study populations rather than inception timeframe alone.

Among 14,889 participants with 353 myocardial infarction events during 61,086 person-years of follow-up in nine studies, the pooled annual rate of myocardial infarction was 0.7% (95% CI = 0.5–1.0) with substantial heterogeneity across studies (I^2^ = 83.7%). We found in subgroup analyses that the annual rate of myocardial infarction remained consistent across study settings, inception points, and regions, with only modest and nonsignificant variations (Supplementary Table 8, Supplementary Figures 9 and 10).

### Meta-regression analysis

We performed meta-regression to explore the associations between study-level characteristics (proportion of participants with atrial fibrillation, lobar ICH, hypertension, diabetes, history of ischemic heart disease, and history of ischemic stroke or transient ischemic attack (TIA)) and annual rates of recurrent ICH and ischemic stroke (Supplementary Figures 11–15). We found only one statistically significant association, which was between a higher prevalence of atrial fibrillation and an increased risk of ischemic stroke (Supplementary Figure 16).

### Sensitivity analysis

We performed sensitivity analyses to assess the impact of study quality, follow-up methods, and specific outcome definitions, and found that event rates of recurrent ICH and ischemic stroke were consistent across these factors (Supplementary Tables 9–11).

## Discussion

The major finding of this comprehensive systematic review and meta-analysis was that recurrent ICH and ischemic stroke were the most frequently reported MACE outcomes after ICH. We found comparable annual rates of recurrent ICH and ischemic stroke across studies, with pooled estimates of 2.08 and 2.00 events per 100 person-years, respectively. These findings illustrate the similar risks of both ischemic and hemorrhagic events for ICH survivors. However, evidence about MACE, MACE subtypes, and other MACE components remains limited due to heterogeneity in definitions and infrequent reporting. Although we were unable to generate a pooled analysis of MACE, individual studies consistently reported a high risk after ICH, with rates ranging from 4.2% to 14.7%.

Previous systematic reviews reported annual recurrent ICH rates of 1.3–7.4%,^7^ and 0.9–11.6%,^2^ with higher rates after lobar ICH compared with deep ICH. Our pooled estimate of 2.1% lies within this range, although meta-regression did not show a significant association with the proportion of lobar ICH in each study, likely due to the limited number of included studies. We also confirm the high risk of ischemic events after ICH, consistent with a multicentre population-based study that reported an annual rate of 3.6% for arterial ischemic events and 2.3% for ischemic stroke, showing that ICH survivors have more than twice the risk of ischemic events and over triple the risk of ischemic stroke compared to general population^5^. Our pooled estimate of 2% for ischemic stroke is also consistent with previous systematic reviews, which reported annual rates ranging from 1.4% to 7.4%^7^ and from 0.8% to 3%.^2^

This study has strengths. We used a pre-specified protocol, conducted a comprehensive, systematic search of the literature, unrestricted by language, and sought relevant data from investigators if unavailable in study reports. By including only studies that reported at least one hemorrhagic and one ischemic MACE component, we focused on studies that reported these events in context rather than in isolation. Although we were unable to perform a pooled analysis of MACE, we conducted a pooled analysis of individual MACE components and sensitivity analyses that accounted for variable definitions, study design, and risk of bias to evaluate the consistency of our findings. We included studies across different settings and regions, which allowed us to investigate variability across populations and enhance the generalizability of the findings.

There are some limitations that are primarily due to the characteristics of the included studies. Definitions and methods of ascertainment of MACE and MACE subtypes varied extensively between studies, preventing pooled analysis. Although there is extensive data on ICH and ischemic stroke, extracranial MACE components have been reported infrequently. Our meta-regression analyses had limited power because of the small number of studies that reported specific comorbidities.

The main implication for clinical practice is that the comparable risks of recurrent ICH and ischemic stroke after ICH demonstrate the need for better strategies for secondary prevention of both ischemic and hemorrhagic events. Current clinical approaches focus on reducing the risk of recurrent ICH by blood pressure reduction and lifestyle modification.^30,42–44^ Despite improvements in blood pressure control since the PROGRESS trial was published, annual rates of recurrent stroke after ICH have remained relatively stable over time, and MACE continues to be a common problem after ICH.

In clinical practice, clinicians try to personalize treatment strategies. Our findings about the absolute risks of MACE help them to do this, but risk factors could also help to focus on high-risk groups. Apart from lobar ICH location being a risk factor for recurrent ICH^6,9,45,46^ and our confirmation that atrial fibrillation is a risk factor for ischemic stroke after ICH, other risk factors remain to be identified and confirmed. This uncertainty is intensified because ICH survivors often present with multiple comorbidities, including age, hypertension, diabetes, renal impairment, and a history of stroke,^2,3,11,47^ which can predispose them to ischemic and hemorrhagic MACE. Understanding the associations of these risk factors—alone or in combination—could improve prediction of MACE subtypes. Clinicians need to be able to consider approaches to preventing ischemic and hemorrhagic MACE, informed by whether these risk factors modify the effects of anticoagulants^48^ and antiplatelet agents.^29,41^

Future research should derive and validate prediction models for MACE outcomes and subtypes after ICH. Multivariable risk models will require individual participant data in collaborative pooled analyses to attain sufficiently large sample sizes to power these analyses and explore generalizability across the world. We are addressing this with an individual participant data meta-analysis involving many of the studies in our systematic review (PROSPERO CRD420251029579). However, whether individual risk factors for MACE outcomes, or a patient’s predicted risk of MACE using multiple variables in a risk prediction model, modify the effects of secondary prevention treatments remains to be determined in subgroup analyses of randomized controlled trials. Ultimately, resolving these uncertainties may permit personalized secondary prevention decisions after ICH, particularly regarding antithrombotic therapy.

In conclusion, risks of recurrent ICH and ischemic stroke are comparable after ICH and seem to be higher than the risk of myocardial infarction, but some uncertainty remains about the risk of MACE overall, risk factors for MACE outcomes, and accurate prediction models for MACE after ICH, all of which should be addressed by future research.

## Supplemental Material

sj-docx-1-wso-10.1177_17474930261416692 – Supplemental material for Rates and risk factors for major adverse cardiovascular and cerebrovascular events after stroke due to intracerebral hemorrhage: Systematic review and study-level meta-analysis Supplemental material, sj-docx-1-wso-10.1177_17474930261416692 for Rates and risk factors for major adverse cardiovascular and cerebrovascular events after stroke due to intracerebral hemorrhage: Systematic review and study-level meta-analysis by Vega Pratiwi Putri, Neshika Samarasekera, Tom J Moullaali, Saketh Jampana, Joseph Aked, Signild Åsberg, Sam Schulman, Georgios Tsivgoulis, Maria Pikilidou, Hsin-Hsi Tsai, Li-Kai Tsai, Phyo Kyaw Myint, Tiberiu A Pana, Charlotte Cordonnier, Barbara Casolla, David Gaist, Alessandro Pezzini, Pol Camps-Renom, Catharina J.M. Klijn, Michele Romoli, Arnstein Tveiten, Ming Liu, Mangmang Xu, Bo Wu, David Werring, Philip S Nash, Gargi Banerjee, Linxin Li and Rustam Al-Shahi Salman in International Journal of Stroke

## References

[1] Feigin VL Abate MD Abate YH , et al. Global, regional, and national burden of stroke and its risk factors, 1990–2021: a systematic analysis for the Global Burden of Disease Study 2021. Lancet Neurol 2024; 23: 973–1003. 39304265 10.1016/S1474-4422(24)00369-7 PMC12254192

[2] Li L Murthy SB. Cardiovascular events after intracerebral hemorrhage. Stroke 2022; 53: 2131–2141. 35674043 10.1161/STROKEAHA.122.036884 PMC9247019

[3] Gaist D Hald SM García Rodríguez LA , et al. Association of prior intracerebral hemorrhage with major adverse cardiovascular events. JAMA Netw Open 2022; 5: e2234215. 10.1001/jamanetworkopen.2022.34215 PMC9530971 36190733

[4] Chen Y Wright N Guo Y , et al. Mortality and recurrent vascular events after first incident stroke: a 9-year community-based study of 0.5 million Chinese adults. Lancet Glob Health 2020; 8: e580–e590. 10.1016/S2214-109X(20)30069-3 PMC7090905 32199124

[5] Murthy SB Zhang C Diaz I , et al. Association between intracerebral hemorrhage and subsequent arterial ischemic events in participants from 4 population-based cohort studies. JAMA Neurol 2021; 78: 809–816. 33938907 10.1001/jamaneurol.2021.0925 PMC8094038

[6] Li L Poon MTC Samarasekera NE , et al. Risks of recurrent stroke and all serious vascular events after spontaneous intracerebral haemorrhage: pooled analyses of two population-based studies. Lancet Neurol 2021; 20: 437–447. 34022170 10.1016/S1474-4422(21)00075-2 PMC8134058

[7] Poon MT Fonville AF Al-Shahi Salman R. Long-term prognosis after intracerebral haemorrhage: systematic review and meta-analysis. J Neurol Neurosurg Psychiatry 2014; 85: 660–667. 24262916 10.1136/jnnp-2013-306476

[8] Murthy SB Diaz I Wu X , et al. Risk of arterial ischemic events after intracerebral hemorrhage. Stroke 2020; 51: 137–142. 31771458 10.1161/STROKEAHA.119.026207 PMC7001742

[9] Casolla B Moulin S Kyheng M , et al. Five-year risk of major ischemic and hemorrhagic events after intracerebral hemorrhage. Stroke 2019; 50: 1100–1107. 31009357 10.1161/STROKEAHA.118.024449

[10] Tveiten A Ljøstad U Mygland Å Naess H. Leukoaraiosis is associated with short- and long-term mortality in patients with intracerebral hemorrhage. J Stroke Cerebrovasc Dis 2013; 22: 919–925. 23433781 10.1016/j.jstrokecerebrovasdis.2013.01.017

[11] Pezzini A Iacoviello L Di Castelnuovo A , et al. Long-term risk of arterial thrombosis after intracerebral hemorrhage: MUCH-Italy. Stroke 2024; 55: 634–642. 38299371 10.1161/STROKEAHA.123.044626 PMC10896192

[12] Shamseer L Moher D Clarke M , et al. Preferred reporting items for systematic review and meta-analysis protocols (PRISMA-P) 2015: elaboration and explanation. BMJ 2015; 349: g7647. 10.1136/bmj.g7647 25555855

[13] PROGRESS Collaborative Group. Randomised trial of a perindopril-based blood-pressure-lowering regimen among 6,105 individuals with previous stroke or transient ischaemic attack. Lancet 2001; 358: 1033–1041. 11589932 10.1016/S0140-6736(01)06178-5

[14] Arima H Anderson C Omae T , et al. Degree of blood pressure reduction and recurrent stroke: the PROGRESS trial. J Neurol Neurosurg Psychiatry 2014; 85: 1284–1285. 24828894 10.1136/jnnp-2014-307856

[15] Cochrane A Chen C Stephen J , et al. Antithrombotic treatment after stroke due to intracerebral haemorrhage. Cochrane Database Syst Rev 2023; 1: CD012144. 10.1002/14651858.CD012144.pub3 PMC9878977 36700520

[16] Schwarzer G Carpenter JR Rücker G. Meta-Analysis with R. Cham: Springer, 2015.

[17] Spittal MJ Pirkis J Gurrin LC. Meta-analysis of incidence rate data in the presence of zero events. BMC Med Res Methodol 2015; 15: 42. 25925169 10.1186/s12874-015-0031-0 PMC4422043

[18] Yan F Yi Z Hua Y , et al. Predictors of mortality and recurrent stroke within five years of intracerebral hemorrhage. Neurol Res 2018; 40: 466–472. 30134784 10.1080/01616412.2018.1451266

[19] Lin FJ Jhang JG Kuo YH , et al. Cardiovascular event recurrence and costs after first myocardial infarction, ischemic stroke, or intracerebral hemorrhage in Taiwan. Acta Cardiol Sin 2023; 39: 457–468. 37229340 10.6515/ACS.202305_39(3).20221021A PMC10203728

[20] Majeed A Kim YK Roberts RS Holmström M Schulman S. Optimal timing of resumption of warfarin after intracranial hemorrhage. Stroke 2010; 41: 2860–2866. 21030703 10.1161/STROKEAHA.110.593087

[21] Aked J Delavaran H Lindgren AG. Survival, causes of death and recurrence up to 3 years after stroke: a population-based study. Eur J Neurol 2021; 28: 4060–4068. 34327786 10.1111/ene.15041

[22] Åsberg S Farahmand B Henriksson KM Appelros P. Statins as secondary preventives in patients with intracerebral hemorrhage. Int J Stroke 2020; 15: 61–68. 30484749 10.1177/1747493018816476

[23] Weimar C Benemann J Terborg C , et al. Recurrent stroke after lobar and deep intracerebral hemorrhage: a hospital-based cohort study. Cerebrovasc Dis 2011; 32: 283–288. 21893981 10.1159/000330643

[24] Sembolini A Romoli M Pannacci U , et al. Acute hematoma expansion after spontaneous intracerebral hemorrhage: risk factors and impact on long-term prognosis. Neurol Sci 2020; 41: 2503–2509. 32215850 10.1007/s10072-020-04356-y

[25] Tsivgoulis G Katsanos AH Patousi A , et al. Stroke recurrence and mortality in northeastern Greece: the Evros stroke registry. J Neurol 2018; 265: 2379–2387. 30128708 10.1007/s00415-018-9005-6

[26] Moliner-Abós C Albertí-Vall B Millan-Álvarez X , et al. Left atrial appendage occlusion in patients with spontaneous intracerebral hemorrhage: an observational study. J Stroke Cerebrovasc Dis 2024; 33: 107481. 38064973 10.1016/j.jstrokecerebrovasdis.2023.107481

[27] Schreuder FHBM van Nieuwenhuizen KM Hofmeijer J , et al. Apixaban versus no anticoagulation after anticoagulation-associated intracerebral haemorrhage in patients with atrial fibrillation in the Netherlands (APACHE-AF): a randomised, open-label, phase 2 trial. Lancet Neurol 2021; 20: 907–916. 34687635 10.1016/S1474-4422(21)00298-2

[28] SoSTART Collaboration. Effects of oral anticoagulation for atrial fibrillation after spontaneous intracranial haemorrhage in the UK: a randomised, open-label, assessor-masked, pilot-phase, non-inferiority trial. Lancet Neurol 2021; 20: 842–853. 34487722 10.1016/S1474-4422(21)00264-7

[29] Al- Shahi Salman R Dennis MS Sandercock PAG , et al. Effects of antiplatelet therapy after stroke caused by intracerebral hemorrhage: extended follow-up of the RESTART randomized clinical trial. JAMA Neurol 2021; 78: 1179. 34477823 10.1001/jamaneurol.2021.2956 PMC8417806

[30] Teo KC Keins S Abramson JR , et al. Blood pressure control targets and risk of cardiovascular and cerebrovascular events after intracerebral hemorrhage. Stroke 2023; 54: 78–86. 36321455 10.1161/STROKEAHA.122.039709

[31] Moon JY Lee JG Kim JH. Antiplatelet therapy after intracerebral hemorrhage and subsequent clinical events: a 12-year South Korean cohort study. Eur Neurol 2021; 84: 183–191. 33831859 10.1159/000514552

[32] Ma X Liu D Niu S , et al. Low-dose antiplatelet therapy survey after intracerebral hemorrhage in China: a retrospective hospital-based study. Neurosurg Rev 2021; 44: 2923–2931. 33502641 10.1007/s10143-021-01483-8

[33] Xu M Li B Zhong D , et al. Cerebral small vessel disease load predicts functional outcome and stroke recurrence after intracerebral hemorrhage: a median follow-up of 5 years. Front Aging Neurosci 2021; 13: 628271. 33679377 10.3389/fnagi.2021.628271 PMC7933464

[34] Xu T Feng Y Wu W , et al. The predictive values of different small vessel disease scores on clinical outcomes in mild ICH patients. J Atheroscler Thromb 2021; 28: 997–1008. 33551444 10.5551/jat.61267 PMC8532058

[35] Ou R Tang YM Li F. The influence of enlarged perivascular spaces on the prognosis of patients with intracerebral hemorrhage [Chinese]. Chin J Contemp Neurol Neurosurg 2018; 18: 807–812.

[36] Szlachetka WA Pana TA Tiamkao S , et al. Impact of diabetes on complications, long term mortality and recurrence in 608,890 hospitalised patients with stroke. Glob Heart 2020; 15: 2. 32489775 10.5334/gh.364 PMC7218766

[37] Tsai HH Chen SJ Tsai LK , et al. Long-term vascular outcomes in patients with mixed location intracerebral hemorrhage and microbleeds. Neurology 2021; 96: e995–e1004. 10.1212/WNL.0000000000011378 33361256

[38] Chen CH Chu YT Chen YF , et al. Comparison of clinical and neuroimaging features between NOTCH3 mutations and nongenetic spontaneous intracerebral haemorrhage. Eur J Neurol 2022; 29: 3243–3254. 35781912 10.1111/ene.15485

[39] Banerjee G Wilson D Ambler G , et al. Longer term stroke risk in intracerebral haemorrhage survivors. J Neurol Neurosurg Psychiatry 2020; 91: 840–845. 32554800 10.1136/jnnp-2020-323079

[40] Jung NY Cho J. Clinical effects of restarting antiplatelet therapy in patients with intracerebral hemorrhage. Clin Neurol Neurosurg 2022; 220: 107361. 35835024 10.1016/j.clineuro.2022.107361

[41] RESTART Collaboration. Effects of antiplatelet therapy after stroke due to intracerebral haemorrhage (RESTART): a randomised, open-label trial. Lancet 2019; 393: 2613–2623. 31128924 10.1016/S0140-6736(19)30840-2 PMC6617509

[42] Intercollegiate Stroke Working Party. National clinical guideline for stroke for the UK and Ireland. National Clinical Guideline for Stroke. Available at: https://www.strokeguideline.org/ (2023, accessed 18 March 2025).

[43] Sandset EC Anderson CS Bath PM , et al. European Stroke Organisation (ESO) guidelines on blood pressure management in acute ischaemic stroke and intracerebral haemorrhage. Eur Stroke J 2021; 6: XLVIII–LXXXIX. 10.1177/23969873211012133 PMC8370078 34780578

[44] Steiner T Purrucker JC Aguiar de Sousa D , et al. European Stroke Organisation (ESO) and European Association of Neurosurgical Societies (EANS) guideline on stroke due to spontaneous intracerebral haemorrhage. Eur Stroke J 2025; 10: 1007–1086. 40401775 10.1177/23969873251340815 PMC12098356

[45] Li L Luengo-Fernandez R Zuurbier SM , et al. Ten-year risks of recurrent stroke, disability, dementia and cost in relation to site of primary intracerebral haemorrhage: population-based study. J Neurol Neurosurg Psychiatry 2020; 91: 580–585. 32165376 10.1136/jnnp-2019-322663 PMC7279204

[46] Boe NJ Hald SM Jensen MM , et al. Major cardiovascular events after spontaneous intracerebral hemorrhage by hematoma location. JAMA Netw Open 2023; 6: e235882. 10.1001/jamanetworkopen.2023.5882 PMC10077102 37017964

[47] McGrath ER Kapral MK Fang J , et al. Which risk factors are more associated with ischemic stroke than intracerebral hemorrhage in patients with atrial fibrillation? Stroke 2012; 43: 2048–2054. 22618379 10.1161/STROKEAHA.112.654145

[48] Al-Shahi Salman R Stephen J Tierney JF , et al. Effects of oral anticoagulation in people with atrial fibrillation after spontaneous intracranial haemorrhage (COCROACH): prospective, individual participant data meta-analysis of randomised trials. Lancet Neurol 2023; 22: 1140–1149. 37839434 10.1016/S1474-4422(23)00315-0

