# Supplementary material for: Rates and risk factors for major adverse cardiovascular and cerebrovascular events after stroke due to intracerebral hemorrhage: Systematic review and study-level meta-analysis
Source: Int J Stroke. 2026 Jan 28;21(6):778–87. doi: 10.1177/17474930261416692 (PMC13291413; doi:10.1177/17474930261416692)
Supplement: sj-docx-1-wso-10.1177_17474930261416692 – Supplemental material for Rates and risk factors for major adverse cardiovascular and cerebrovascular events after stroke due to intracerebral hemorrhage: Systematic review and study-level meta-analysis [file sj-docx-1-wso-10.1177_17474930261416692.docx]

**Supplemental Material**

Rates and Risk Factors for Major Adverse Cardiovascular and Cerebrovascular Events after stroke due to intracerebral haemorrhage: systematic review and study-level meta-analysis

[**Supplementary table 1:** Ovid MEDLINE(R) electronic search strategies 2](#_Toc216169780)

[**Supplementary table 2:** EMBASE electronic search strategies 3](#_Toc216169781)

[**Supplementary table 3:** Clinical trial database electronic search strategies 4](#_Toc216169782)

[**Supplementary figure 1:** PRISMA Flow chart 5](#_Toc216169783)

[**Supplementary figure 2:** Risk of bias assessment summary plot 6](#_Toc216169784)

[**Supplementary figure 3**: Risk of bias in individual studies 6](#_Toc216169785)

[**Supplementary table 4:** Included studies’ characteristics 7](#_Toc216169786)

[**Supplementary table 5:** Study-specific definitions of the composite outcome of Major Adverse Cardiovascular and Cerebrovascular Event (MACE) 8](#_Toc216169787)

[**Supplementary table 6:** Study-specific definitions of ischaemic Major Adverse Cardiovascular and Cerebrovascular Event (MACE) 9](#_Toc216169788)

[**Supplementary table 7:** Study-specific definitions of haemorrhagic Major Adverse Cardiovascular and Cerebrovascular Event (MACE) 10](#_Toc216169789)

[**Supplementary table 8:** Annual rates of recurrent intracerebral haemorrhage, ischaemic stroke, and myocardial infarction, with sub-group analyses by setting, inception point, and region 11](#_Toc216169790)

[**Supplementary figure 4:** Summary sub-group’pooled rate of recurrent ICH, ischaemic stroke, and myocardial infraction 12](#_Toc216169791)

[**Supplementary figure 5**: Forest plot of the annual rate of recurrent ICH sub-grouped by region. 13](#_Toc216169792)

[**Supplementary figure 6**: Forest plot of the annual rate of recurrent ICH sub-grouped by inception point 14](#_Toc216169793)

[**Supplementary figure 7:** Forest plot annual rate of ischaemic stroke sub-grouped by region. 15](#_Toc216169794)

[**Supplementary figure 8:** Forest plot of the annual rate of ischaemic stroke sub-grouped by inception point 16](#_Toc216169795)

[**Supplementary figure 9**: Forest plot of the annual rate of myocardial infarction sub-grouped by inception point 17](#_Toc216169796)

[**Supplementary figure 10**: Forest plot annual rate of myocardial infarction sub-grouped by region. 18](#_Toc216169797)

[**Supplementary figure 11:** Meta-regression analysis of the study-level estimate of outcome event rates on the study-level proportion of participants with lobar intracerebral haemorrhage 19](#_Toc216169798)

[**Supplementary figure 12**: Meta-regression analysis of the study-level estimate of outcome event rates on the study-level proportion of participant with hypertension 19](#_Toc216169799)

[**Supplementary figure 13**: Meta-regression analysis of the study-level estimate of outcome event rates on the study-level proportion of participant with diabetes mellitus 20](#_Toc216169800)

[**Supplementary figure 14**: Meta-regression analysis of the study-level estimate of outcome event rates on the study-level proportion of participant with ischaemic heart disease 20](#_Toc216169801)

[**Supplementary figure 15:** Meta-regression analysis of the study-level estimate of outcome event rates on the study-level proportion of participant with prior ischaemic stroke or transient ischaemic attack 21](#_Toc216169802)

[**Supplementary figure 16:** Meta-regression analysis of the study-level estimate of outcome event rates on the study-level proportion of participant with atrial fibrillation 21](#_Toc216169803)

[**Supplementary table 9**: Sensitivity analysis by studies’ risk of bias and follow-up methods 22](#_Toc216169804)

[**Supplementary table 10:** Sensitivity analysis by studies’ specific definition of intracerebral haemorrhage 23](#_Toc216169805)

[**Supplementary table 11**: Sensitivity analysis by studies’ specific definition of ischaemic stroke 23](#_Toc216169806)

[Supplementary table 12: PRISMA Checklist 27](#_Toc216169807)

### **Supplementary table 1:** Ovid MEDLINE(R) electronic search strategies

| **#** | **Query** | **Results** |
| --- | --- | --- |
| 1 | ((brain$ or cerebr$ or cerebell$ or intracerebral or intracran$ or parenchymal or intraparenchymal or intraventricular or infratentorial or supratentorial or basal gangli$ or putaminal or putamen or posterior fossa or hemispher$ or stroke or apoplex$) adj5 (h?emorrhag$ or h?ematoma$ or bleed$)).tw. or intracranial hemorrhages/ or cerebral hemorrhage/ or exp basal ganglia hemorrhage/ or cerebral intraventricular hemorrhage/ or intracranial hemorrhage, hypertensive/ or hemorrhagic stroke/ | 92,908 |
| 2 | cohort.mp. or exp cohort studies/ or longitudinal.mp. or follow-up.mp. or exp follow-up studies/ or exp longitudinal studies/ or exp prospective studies/ or prognos*.mp. | 3,947,150 |
| 3 | exp animals/ not humans.sh. | 5,198,374 |
| 4 | (major adverse cardiovascular even* or major adverse cardiac even* or MACE or MACEs or (serious vascular even* or arterial isch?mic even* or major isch?mic even* or serious cardiovascular even*) or arterial thrombosis or (recurren* adj5 (strok* or intracerebral h?morrhag* or ICH))).tw. | 31,073 |
| 5 | (major ischemic adj5 hemorrhagic even*).tw. | 6 |
| 6 | (cardiovascular even* or cardiac even*).mp. | 63,361 |
| 7 | (major adverse adj (cardiac and cerebrovascular) adj even*).tw. | 1,361 |
| 8 | macce.tw. | 1,675 |
| 9 | 4 or 5 or 6 or 7 or 8 | 80,742 |
| 10 | 1 and 2 and 9 | 1,681 |
| 11 | 10 not 3 | 1,681 |
| 12 | limit 11 to yr="2001 -Current" | 1,586 |

### **Supplementary table 2:** EMBASE electronic search strategies

| **#** | **Query** | **Results** |
| --- | --- | --- |
| 1 | basal ganglion hemorrhage/ or brain hemorrhage/ or brain ventricle hemorrhage/ or cerebellum hemorrhage/ | 138,021 |
| 2 | ((brain$ or cerebr$ or cerebell$ or intracerebral or intracran$ or parenchymal or intraparenchymal or intraventricular or infratentorial or supratentorial or basal gangli$ or putaminal or putamen or posterior fossa or hemispher$) adj5 (h?emorrhag$ or h?ematoma$ or bleed$)).tw. | 112,287 |
| 3 | ((h?emorrhag$ or bleed$) adj5 (stroke or apoplex$)).tw. | 34,590 |
| 4 | (ICH or ICHs).tw. | 33,111 |
| 5 | 1 or 2 or 3 or 4 | 205,242 |
| 6 | (Cohort stud* or longitudinal stud* or follow-up).tw. or exp follow up/ or exp prospective studiy/ or prognos*.tw. or exp prognosis/ | 4,181,946 |
| 7 | exp animals/ or exp invertebrate/ or animal experiment/ or animal model/ or animal tissue/ or animal cell/ or nonhuman/ | 32,600,910 |
| 8 | human/ or normal human/ or human cell/ | 25,743,095 |
| 9 | 7 not 8 | 6,907,740 |
| 10 | exp major adverse cardiac event/ or major adverse cardiac even*.tw. or major adverse cardiovascular even*.tw. or MACE.tw. or (serious vascular even* or arterial isch?mic even* or major isch?mic even* or serious cardiovascular even*).tw. or arterial thrombosis.tw. | 54,609 |
| 11 | (recurren* adj5 (strok* or intracerebral h?morrhag* or ICH)).tw. | 17,014 |
| 12 | (major ischemic adj5 hemorrhagic even*).tw. | 10 |
| 13 | (cardiovascular even* or cardiac even*).mp. | 128,687 |
| 14 | (major adverse adj (cardiac and cerebrovascular) adj even*).tw. | 2,660 |
| 15 | macce.tw. | 4,307 |
| 16 | 10 or 11 or 12 or 13 or 14 or 15 | 164,535 |
| 17 | 5 and 6 and 16 | 3,780 |
| 18 | 17 not 9 | 3,772 |
| 19 | limit 18 to yr="2001 -Current" | 3,698 |

### **Supplementary table 3:** Clinical trial database electronic search strategies

| **#** | **Query** | **Results** |
| --- | --- | --- |
| 1 | Database clinical trial: [https://clinicaltrials.gov](https://eur02.safelinks.protection.outlook.com/?url=https%3A%2F%2Fclinicaltrials.gov%2F&data=05%7C02%7C%7C32b64c1966d34d3c688108dc73e399cb%7C2e9f06b016694589878910a06934dc61%7C0%7C0%7C638512668578779470%7CUnknown%7CTWFpbGZsb3d8eyJWIjoiMC4wLjAwMDAiLCJQIjoiV2luMzIiLCJBTiI6Ik1haWwiLCJXVCI6Mn0%3D%7C0%7C%7C%7C&sdata=3i2VlMbW1vUelzb7rgFhycR20BwJXnPEJ2Ka7g3lRAc%3D&reserved=0)  CONDITION/DISEASE: **“**intracerebral hemorrhage” OR “Cerebral Hemorrhage” OR “Intracerebral haemorrhage” OR “intracranial hemorrhage” OR “intracranial haemorrhage” OR “hemorrhagic stroke” OR “cerebral hemorrhage” OR “cerebral haemorrhage  OTHER ITEMS: “stroke recurrence” OR “recurrent stroke” OR “MACE” or “major adverse cardiac event” or “major adverse cardiovascular event” or “Major Adverse Cardiovascular and Cerebrovascular Events” or “MACCE” | 87 |

### **Supplementary figure 1:** PRISMA Flow chart


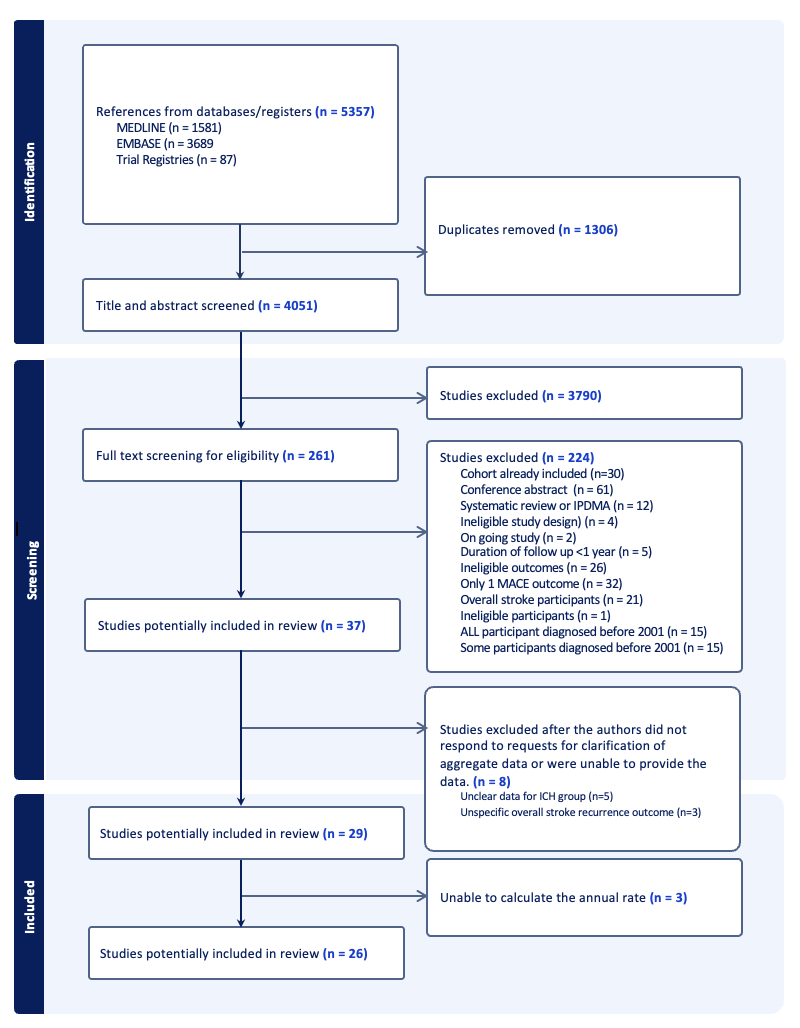


### **Supplementary figure 2:** Risk of bias assessment summary plot


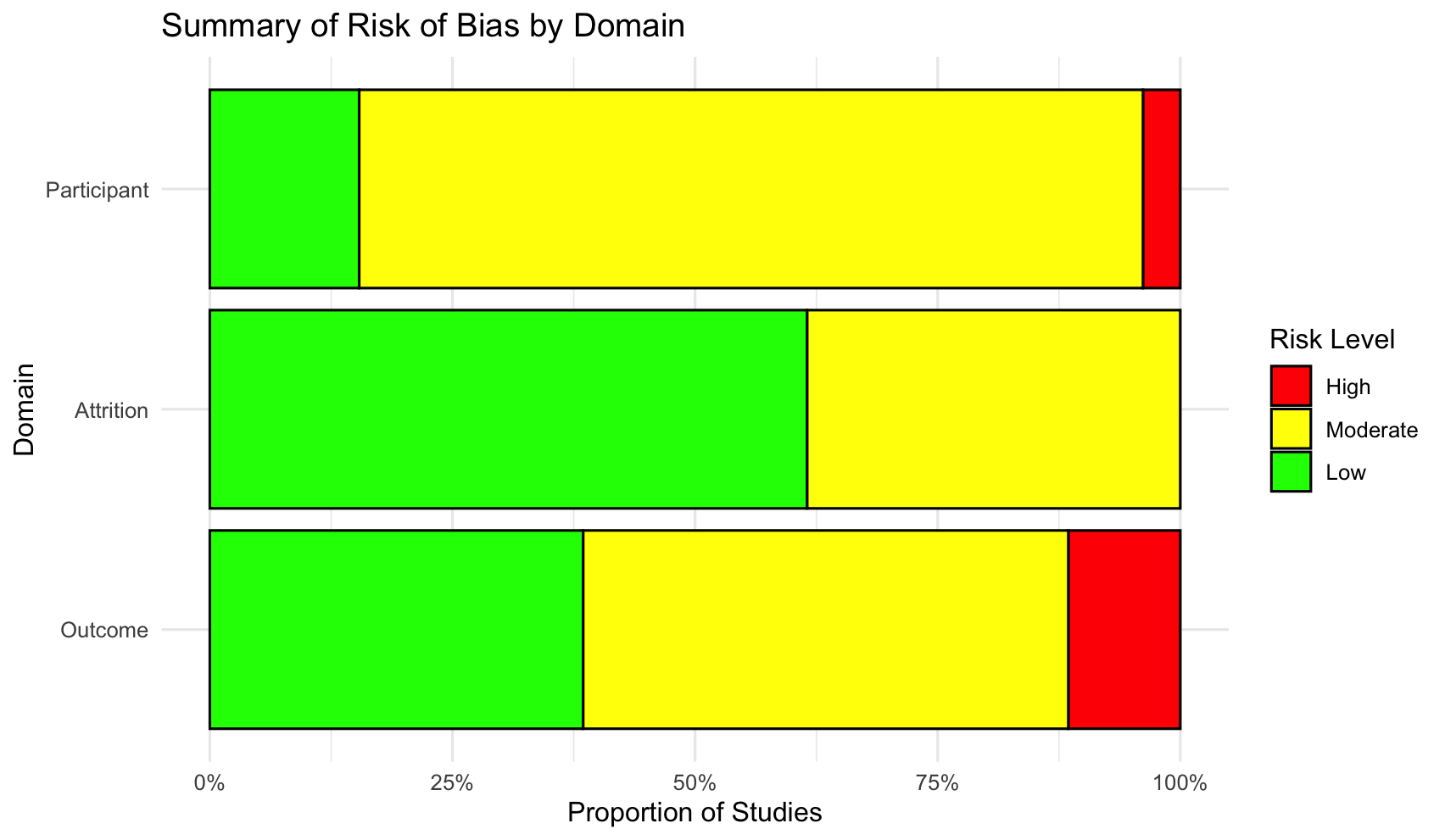


### **Supplementary figure 3**: Risk of bias in individual studies

**
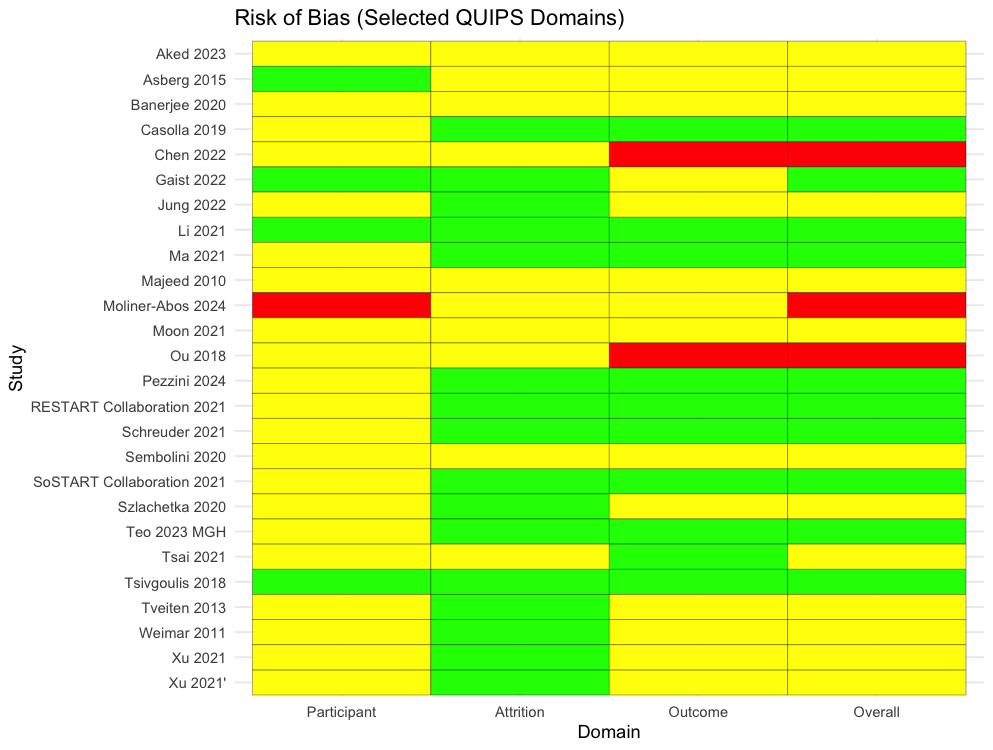
**

### **Supplementary table 4:** Included studies’ characteristics

| **Study** | **Country** | **N** | **Mid-year** | **Median follow-up (years)** | **Study design** | **Setting** | **Ascertainment** | **Follow-up** | **Inception point** | **Median Age** | **Male**  **(%)** | **AF**  **(%)** | **HTN**  **(%)** | **DM**  **(%)** | **IHD**  **(%)** | **History of IS or TIA**  **(%)** |
| --- | --- | --- | --- | --- | --- | --- | --- | --- | --- | --- | --- | --- | --- | --- | --- | --- |
| Weimar ^1^ | Germany | 496 | 2004 | 1.9 | Cohort | Hospital | Prospective | Prospective | Survived at hospital discharge | 70 | 58 | 11 | 82 | 23 | 17 | NA |
| Majeed ^2^ | Sweden, Canada | 70 | 2005 | 1 | Cohort | Hospital | Retrospective | Retrospective | Survived at day-7 | 75 | 57 | 59 | NA | NA | NA | NA |
| Casolla ^3^ | France | 310 | 2006 | 6 | Cohort | Hospital | Prospective | Prospective | Survived at day-30 | 70 | 55 | 11 | 65 | 15 | 15 | 13 |
| Tveiten ^4^ | Norway | 134 | 2007 | 4.7 | Cohort | Hospital | Retrospective | Retrospective | Survived at day-30 | 75 | 55 | 26 | 54 | 13 | 15 | 21 |
| Asberg ^5^ | Sweden | 6,082 | 2007 | 3.1 | Cohort | Hospital | Retrospective | Prospective | Survived at day-30 | 70 | 54 | 14 | 42 | 12 | 8 | NA |
| Gaist ^6^ | Denmark | 8,991 | 2007 | 4.3 | Cohort | Hospital | Retrospective | Retrospective | Survived at day-30 | 71 | 54 | 15 | 68 | 12 | 4 | 14 |
| Moon ^7^ | Korea | 1,007 | 2008 | 2.5 | Cohort | Hospital | Retrospective | Retrospective | Survived at day-30 | 61 | 53 | NA | 80 | 29 | NA | NA |
| Pezzini ^8^ | Italy | 1,729 | 2008 | 3.6 | Cohort | Hospital | Prospective | Prospective | Survived at day-30 | 74 | 57 | 10 | 74 | 19 | 16 | NA |
| Ma ^9^ | China | 312 | 2009 | 4 | Cohort | Hospital | Retrospective | Prospective | Not stated | 64 | 61 | 18 | 67 | 46 | 21 | NA |
| Szlachetka ^10^ | Thailand | 173,236 | 2010 | 4.8 | Cohort | Hospital | Retrospective | Retrospective | Survived at hospital discharge | 61 | 60 | 2 | 55 | 10 | 1 | NA |
| Li ^11^ | UK (Oxford) | 225 | 2009 | 2.8 | Cohort | Population | Prospective | Prospective | ICH onset | 76 | 56 | 25 | 70 | 16 | 9 | 32 |
| Tsivgoulis ^12^ | Greece | 83 | 2011 | 1 | Cohort | Population | Prospective | Prospective | Not stated | 77 | 57 | 26 | 76.80 | 17 | NA | NA |
| Li ^11^ | UK (Lothian) | 419 | 2011 | 1.9 | Cohort | Population | Prospective | Prospective | ICH onset | 75 | 46 | 22 | 64.92 | 11 | 14 | 23 |
| Teo ^13^ | USA | 1,305 | 2011 | 4.2 | Cohort | Hospital | Prospective | Prospective | Survived at day-90 | 70 | 53 | 16.32 | 78.24 | NA | 20 | 9 |
| Sembolini ^14^ | Italy | 140 | 2013 | 2.3 | Cohort | Hospital | Prospective | Prospective | Survived at hospital discharge | 75 | 59 | NA | 87.14 | NA | NA | NA |
| Banerjee ^15^ | UK | 1,094 | 2013 | 3 | Cohort | Hospital | Prospective | Prospective | Not stated | 73 | 57 | 34.28 | 65.63 | 18 | 16 | 22 |
| Xu ^16^ | China | 131 | 2015 | 4.9 | Cohort | Hospital | Prospective | Prospective | Within 90 days after onset | 61 | 75 | NA | 82.44 | 11 | 6 | NA |
| Teo ^13^ | Hongkong | 523 | 2015 | 2.9 | Cohort | Hospital | Prospective | Prospective | Survived at day-90 | 67 | 62 | 7.27 | 58.32 | NA | 7 | 13 |
| Ou ^17^ | China | 306 | 2015 | 1 | Cohort | Hospital | Prospective | Prospective | Within 48hours admission | 70 | 55 | NA | 70.26 | 41 | 19 | NA |
| Jung ^18^ | Korea | 202 | 2016 | 3.6 | Cohort | Hospital | Retrospective | Retrospective | Not stated | 68 | 50 | 9.41 | 78.22 | 26 | 23 | 54 |
| Xu ^19^ | China | 163 | 2016 | 1 | Cohort | Hospital | Prospective | Prospective | Not stated | 63 | 61 | 3.68 | 80.98 | 17 | NA | NA |
| RESTART Collaboration ^20^ | UK | 536 | 2015 | 3 | RCT | Hospital | Prospective | Prospective | Survived at 24hours | 76 | 67 | 17.16 | 74.81 | 24 | 45 | 30 |
| Aked ^21^ | Sweden | 60 | 2016 | 1.9 | Cohort | Population | Retrospective | Retrospective | Not stated | 75 | 52 | NA | NA | NA | NA | NA |
| Tsai ^22^ | Taiwan | 300 | 2016 | 1.9 | Cohort | Hospital | Prospective | Prospective | Survived at day-30 | 63 | 65 | NA | 89.30 | 27 | NA | NA |
| Moliner-Abos (26) | Spain | 40 | 2018 | 3.9 | Cohort | Hospital | Retrospective | Retrospective | Not stated | 77 | 72 | 100 | 90.00 | 18 | 5 | 30 |
| Schreuder ^24^ | Netherland | 101 | 2017 | 1.9 | RCT | Hospital | Prospective | Prospective | Within 7 to 90 days after ICH | 78 | 55 | 100 | 94.06 | 16 | 12 | 24 |
| Chen ^25^ | Taiwan | 109 | 2019 | 3.6 | Cohort | Hospital | Prospective | Prospective | Not stated | 56 | 63 | NA | 97.25 | 22 | NA | NA |
| SoSTART Collaboration ^26^ | UK | 185 | 2019 | 1.3 | RCT | Hospital | Prospective | Prospective | Survived at 24hours | 79 | 65 | 100 | NA | NA | NA | NA |

*^)^ N: number of participants. Mid-year: The midpoint between the start and end dates of participant recruitment in a cohort study. Inception point: time delay from ICH onset to cohort recruitment. AF: atrial fibrillation. HTN: hypertension. DM: diabetes mellitus. IHD: Ischaemic heart disease. TIA: transient ischaemic attack. Two publications ^11,13^ contributed data from two different cohort. Each cohort was analysed separately, resulting in a total of 28 cohorts from 26 publications included.

### **Supplementary table 5:** Study-specific definitions of the composite outcome of Major Adverse Cardiovascular and Cerebrovascular Event (MACE)

| Study | Definition of all MACE |
| --- | --- |
| SoSTART collaboration ^26^ | recurrent symptomatic spontaneous intracranial haemorrhage, ischaemic stroke, MI, sudden cardiac death, death from another vascular cause, death from an unknown cause |
| RESTART collaboration ^20^ | non-fatal stroke, non-fatal myocardial infarction, vascular death (including sudden death, PE, haemorrhage, and death from an unknown cause) |
| Gaist ^6^ | stroke, non-fatal MI, systemic embolism, or vascular death |
| Moliner-Abos ^23^ | death, non-fatal ICH, non-fatal ischaemic stroke, systemic embolism, or major bleeding |
| Moon ^7^ | nonfatal ICH recurrence, nonfatal ischaemic stroke, nonfatal MI, or all-cause mortality |
| Schreuder ^24^ | non-fatal ischaemic stroke, intracerebral haemorrhage, myocardial infarction or vascular death. |
| Tsai ^22^ | ICH, Ischaemic stroke, death |
| Li (6) | non-fatal stroke or myocardial infarction, or vascular death |

### **Supplementary table 6:** Study-specific definitions of ischaemic Major Adverse Cardiovascular and Cerebrovascular Event (MACE)

Of the eight studies reporting ischaemic MACE, all included ischaemic stroke and myocardial infarction as part of their definition, but some studies also included TIA ^9,14^, venous thromboembolism ^6,20^, and extracranial arterial events, such as acute coronary syndrome ^8,9^, coronary artery disease ^14^, acute limb ischaemia ^3,8^, peripheral arterial occlusion ^18,20^, and any arterial revascularisation procedure ^6,8,20^). One study used ischaemic stroke as the sole component of ischaemic MACE ^22^, but in the studies which differentiated between stroke subtypes, ischaemic stroke was combined with myocardial infarction ^7,20,24^ or systemic embolism ^23^

| Study | Definition of ischaemic MACE |
| --- | --- |
| RESTART Collaboration ^20^ | ischemic stroke, myocardial infarction + mesenteric ischemia, peripheral arterial occlusion, deep vein thrombosis, pulmonary embolism, or carotid/coronary/peripheral arterial revascularisation procedures |
| Casolla ^3^ | ischaemic stroke + systemic arterial vascular events, including acute coronary syndrome, acute limb ischemia, coronary artery revascularization (either coronary artery bypass or percutaneous coronary intervention), severe peripheral artery disease (i.e., symptomatic arterial stenosis or stenosis requiring a revascularisation procedure), and severe internal carotid stenosis requiring a revascularisation procedure. |
| Gaist ^6^ | ischemic stroke, myocardial infarction + unspecified stroke, systemic embolism, revascularization procedures, mesenteric ischemia, venous thromboembolism, or sudden cardiac death |
| Jung ^18^ | ischaemic stroke, myocardial infarction + peripheral arterial diseases |
| Ma ^9^ | Ischaemic stroke + acute coronary syndrome, TIA* |
| Pezzini ^8^ | Ischaemic stroke, myocardial infarction, + acute coronary syndrome other than myocardial infarction, acute limb ischaemia, coronary or peripheral or carotid disease requiring revascularisation |
| Schreuder ^24^ | Ischaemic stroke, myocardial infarction + systemic embolism or pulmonary embolism. |
| Sembolini ^14^ | Ischaemic stroke + TIA*, coronary arterial disease |

*TIA: Transient Ischaemic Attack

### **Supplementary table 7:** Study-specific definitions of haemorrhagic Major Adverse Cardiovascular and Cerebrovascular Event (MACE)

Five studies reported haemorrhagic MACE and applied relatively homogenous criteria, typically including intracranial haemorrhage and major extracranial haemorrhage.

| Study | Definition haemorrhagic MACE |
| --- | --- |
| RESTART Collaboration ^20^ | Intracranial haemorrhage + major extracranial haemorrhage (requiring transfusion or endoscopic treatment or surgery, or resulting in death within 30 days) |
| Casolla (9) | Intracranial haemorrhage + other bleeding from BARC* type > 3 |
| Gaist (3) | Intracranial haemorrhage + extracranial haemorrhage, i.e., GI haemorrhage or other major extracranial haemorrhage (acute bleeding anaemia, haematuria, hemopericardium, oesophageal varicose vein haemorrhage, oesophageal haemorrhage, peritoneal haemorrhage, haemothorax, haemorrhage in bile duct or pancreas, or haemorrhage in spinal cord. |
| Pezzini ^8^ | Intracranial haemorrhage + other bleeding from BARC* type > 3 |
| Schreuder ^24^ | Intracranial haemorrhage + major extracranial haemorrhage following ISTH* criteria |

BARC: Bleeding Academic Research Consortium

ISTH: International Society on Thrombosis and Haemostasis

### **Supplementary table 8:** Annual rates of recurrent intracerebral haemorrhage, ischaemic stroke, and myocardial infarction, with sub-group analyses by setting, inception point, and region

|  | **Recurrent ICH** | | | | | **Ischaemic Stroke** | | | | | **Myocardial Infarction** | | | | |
| --- | --- | --- | --- | --- | --- | --- | --- | --- | --- | --- | --- | --- | --- | --- | --- |
|  | Number of cohorts | n | N | Annual rate (95%CI) | *p* | Number of cohorts | n | N | Annual rate  (95%CI) | *p* | Number of cohorts | n | N | Annual rate (95%CI) | *p* |
| **Overall** | 28 | 10,795 | 198,289 | 2.08  (1.68-2.56) | - | 26 | 5,705 | 197,837 | 1.98  (1.48-2.66) | - | 10 | 353 | 14,889 | 0.66  (0.45-0.95) | - |
| **Setting:** |  |  |  |  | 0.69 |  |  |  |  | 0.40 |  |  |  |  | - |
| Population | 4 | 46 | 787 | 1.48  (0.28-7.88) |  | 4 | 27 | 787 | 1.62  (0.98-2.67) |  |  |  |  |  |  |
| Hospital | 24 | 1,0749 | 197,502 | 2.08  (1.68-2.59) |  | 22 | 5678 | 197,050 | 2.09  (1.51-2.89) |  | 10 | 353 | 14,889 | 0.66 (0.45-0.95) |  |
| **Inception point** |  |  |  |  | 0.02 |  |  |  |  | 0.004 |  |  |  |  | 0.11 |
| <1 month | 9 | 9,405 | 175,613 | 2.61  (2.56-2.66) |  | 9 | 4,408 | 175,473 | 3.17  (1.86-5.40) |  | 2 | 23 | 721 | 1.13  (0.75-1.70) |  |
| >1 month | 9 | 1,262 | 20,381 | 1.75  (1.18-2.61) |  | 8 | 1,173 | 20,381 | 1.28  (1.02-1.60) |  | 6 | 324 | 13,865 | 0.60  (0.37-0.96) |  |
| Unknown | 10 | 128 | 2,295 | 1.90  (1.41-2.56) |  | 9 | 124 | 1,983 | 2.16  (1.27; 3.67) |  | 2 | 6 | 303 | 0.63  (0.28-1.41) |  |
| **Region** |  |  |  |  | 0.37 |  |  |  |  | 0.78 |  |  |  |  | 0.78 |
| Asia | 10 | 9,462 | 176,289 | 2.35  (1.65; 3.33) |  | 9 | 4,365 | 175,977 | 1.88  (1.10-3.18) |  | 3 | 35 | 1,530 | 0.55  (0.11-2.74) |  |
| Other than Asia | 18 | 1,333 | 22,000 | 1.93  (1.50-2.48) |  | 17 | 1,340 | 21,860 | 2.05  (1.45-2.92) |  | 7 | 314 | 13,157 | 0.64  (0.50-0.82) |  |

^*)^Two publications ^11,13^ contributed data from two different cohort. Each cohort was analysed separately, resulting in a total of 28 cohorts from 26 publications for recurrent ICH outcomes, 26 cohorts from 24 publications for ischemic stroke outcomes, and 10 cohorts from 9 publications for myocardial infarction outcomes.

^**)^ n = number of outcome events. N= total number of participants

^***)^ *p*= P-values test between-group heterogeneity within each subgroup category

**Supplementary figure 4:** Summary sub-group’pooled rate of recurrent ICH, ischaemic stroke, and myocardial infraction

**
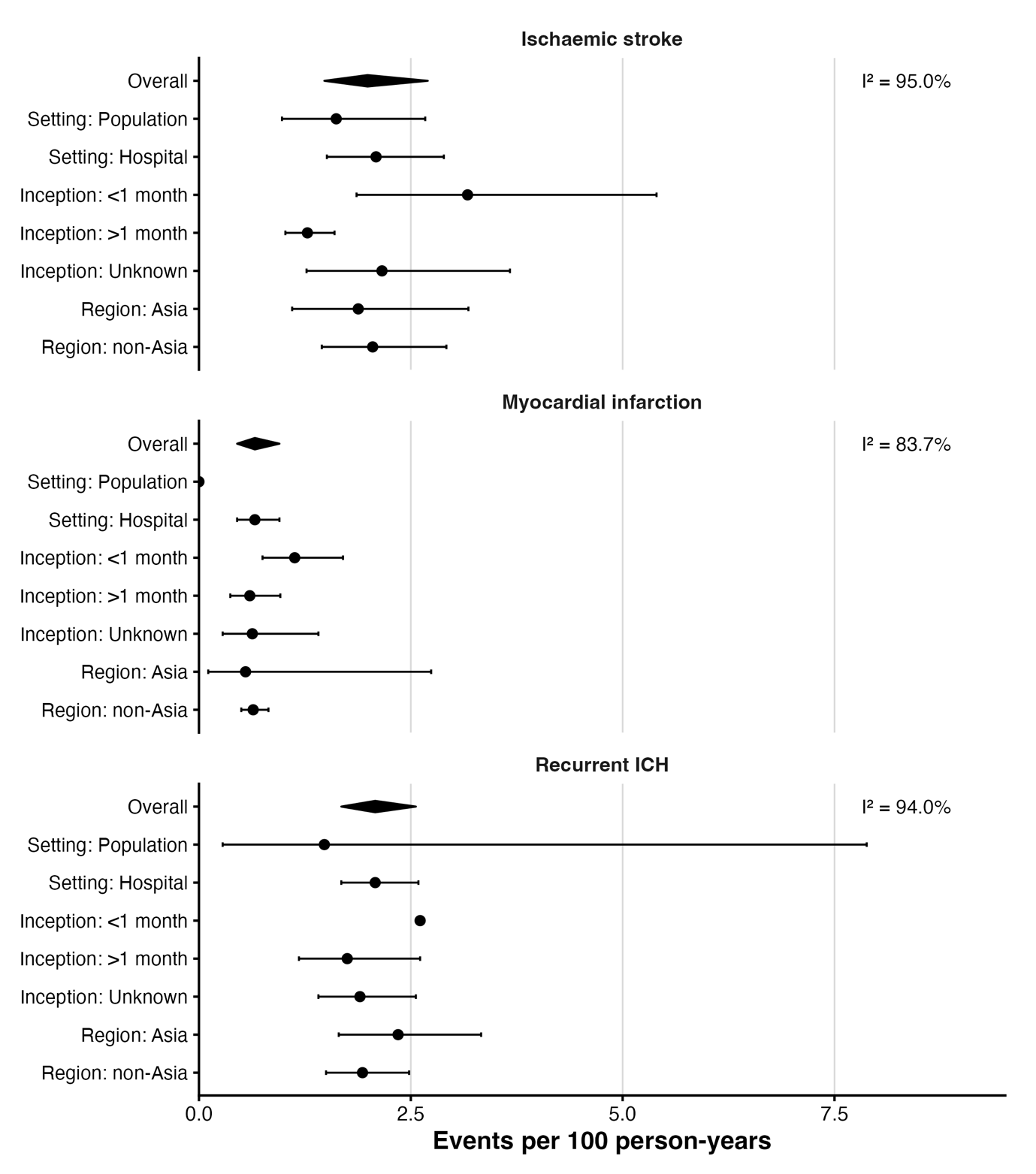
**

**Supplementary figure 5**: Forest plot of the annual rate of recurrent ICH sub-grouped by region. Studies are organized in ascending chronological order of cohort mid-year. Error bars are 95% CIs, and the diamonds represent pooled estimates.


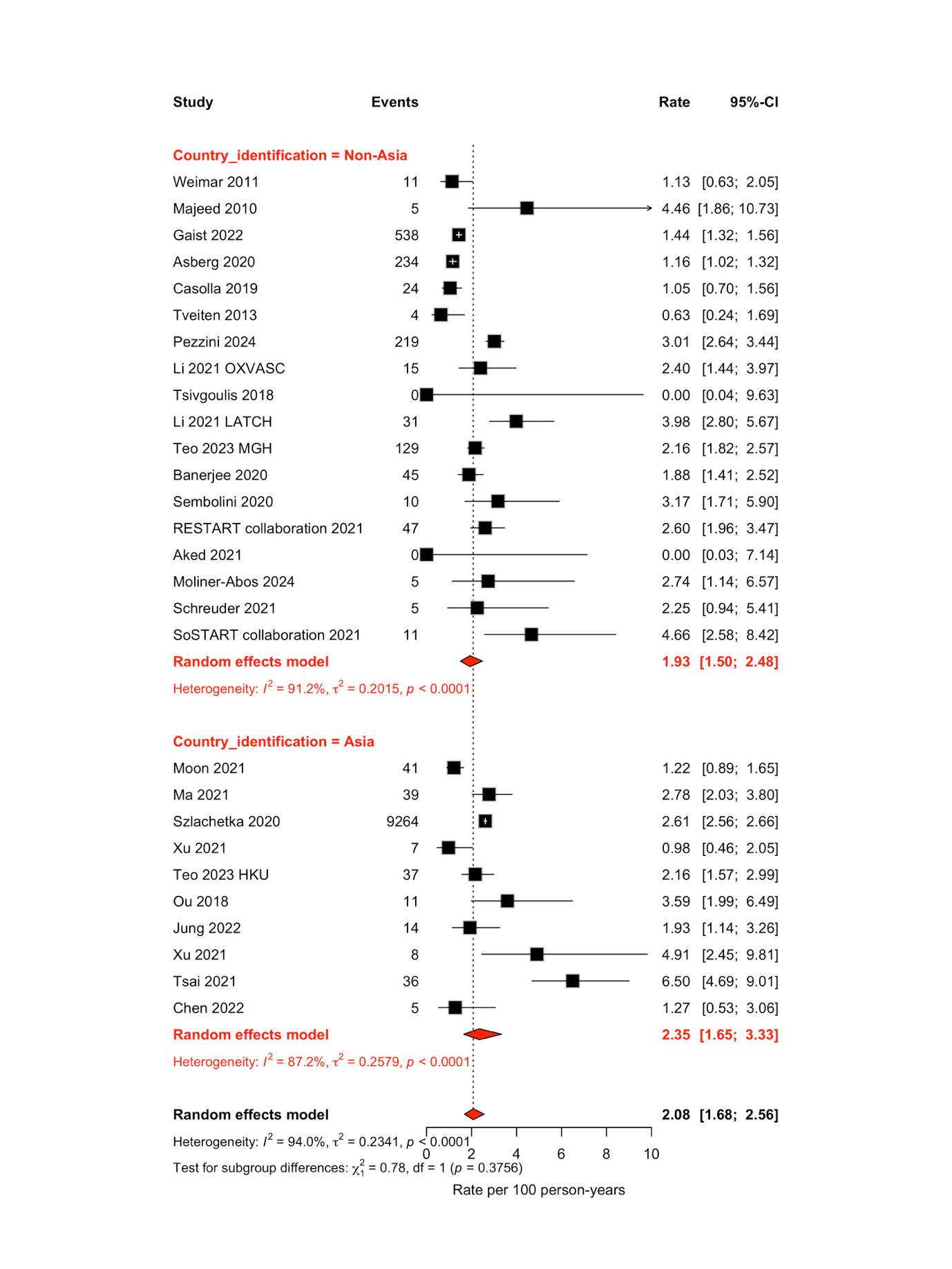


**Supplementary figure 6**: Forest plot of the annual rate of recurrent ICH sub-grouped by inception point. Studies are organized in ascending chronological order of cohort mid-year. Error bars are 95% CIs, and the diamonds represent pooled estimates.


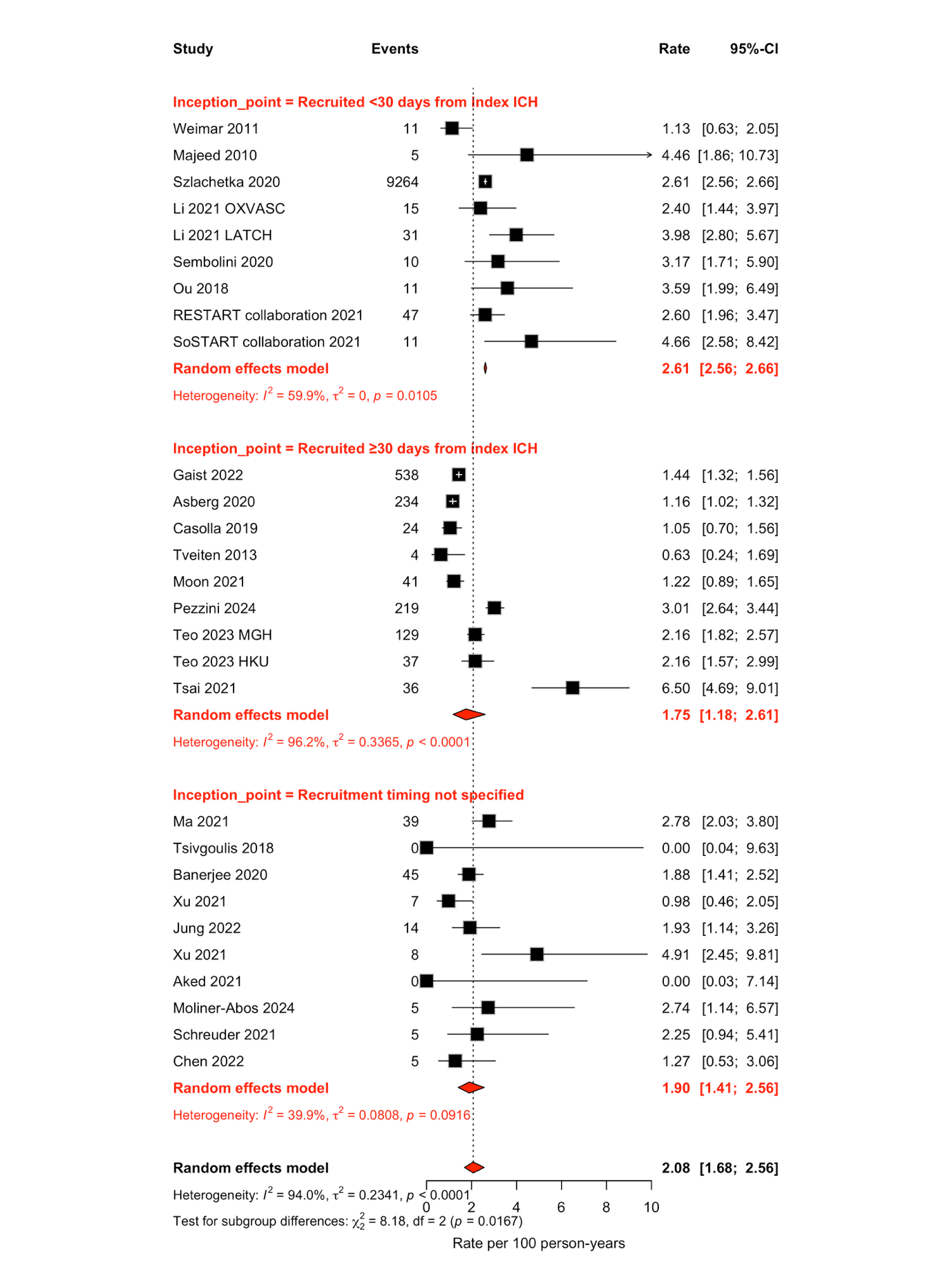


**Supplementary figure 7:** Forest plot annual rate of ischaemic stroke sub-grouped by region. Studies are organized ascending chronological order of cohort mid-year. Error bars are 95% CIs, and the diamonds represent the pooled estimate.


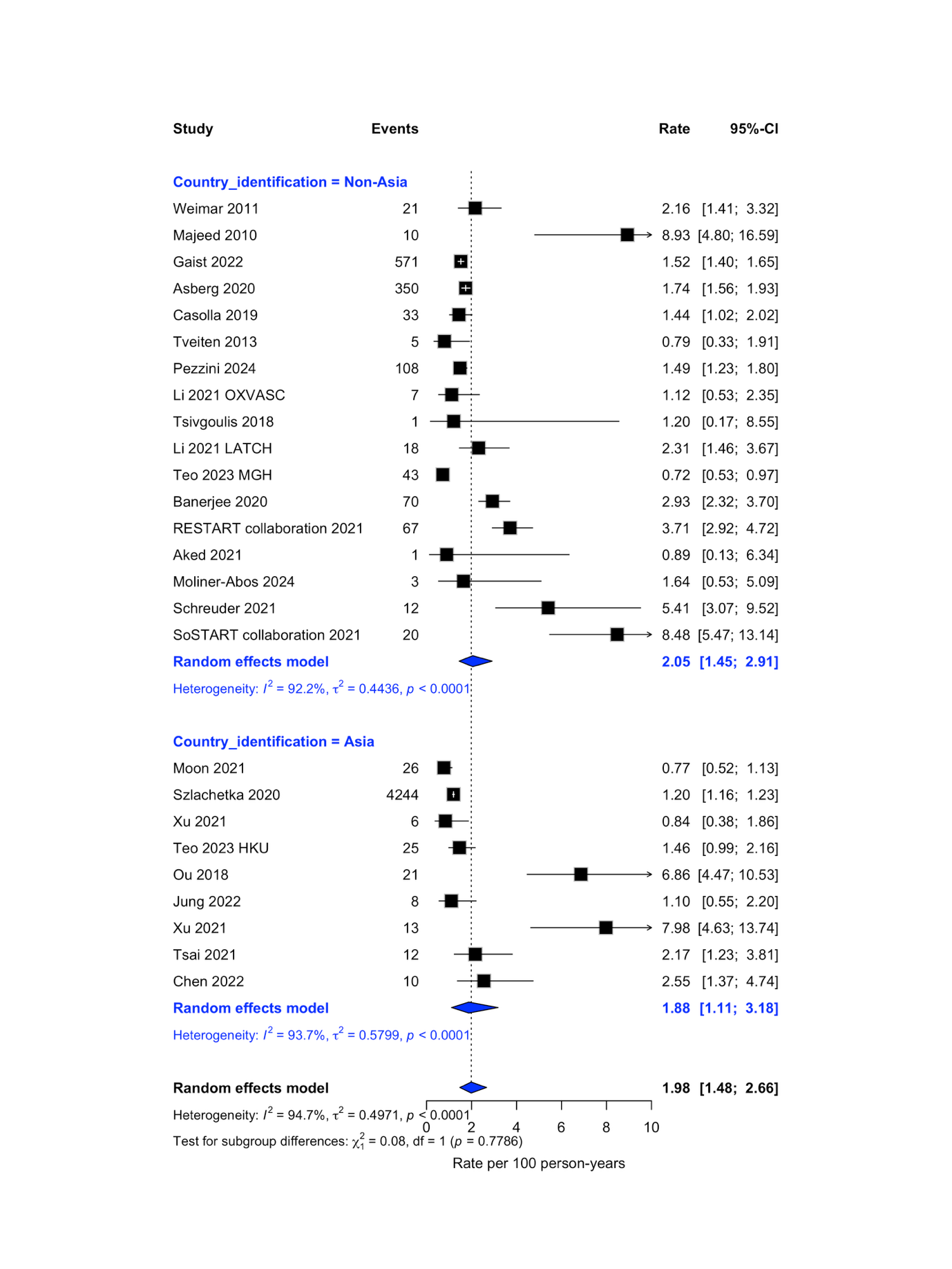


**Supplementary figure 8:** Forest plot of the annual rate of ischaemic stroke sub-grouped by inception point. Studies are organized in ascending chronological order of cohort mid-year. Error bars are 95% CIs, and the diamonds represent pooled estimates.


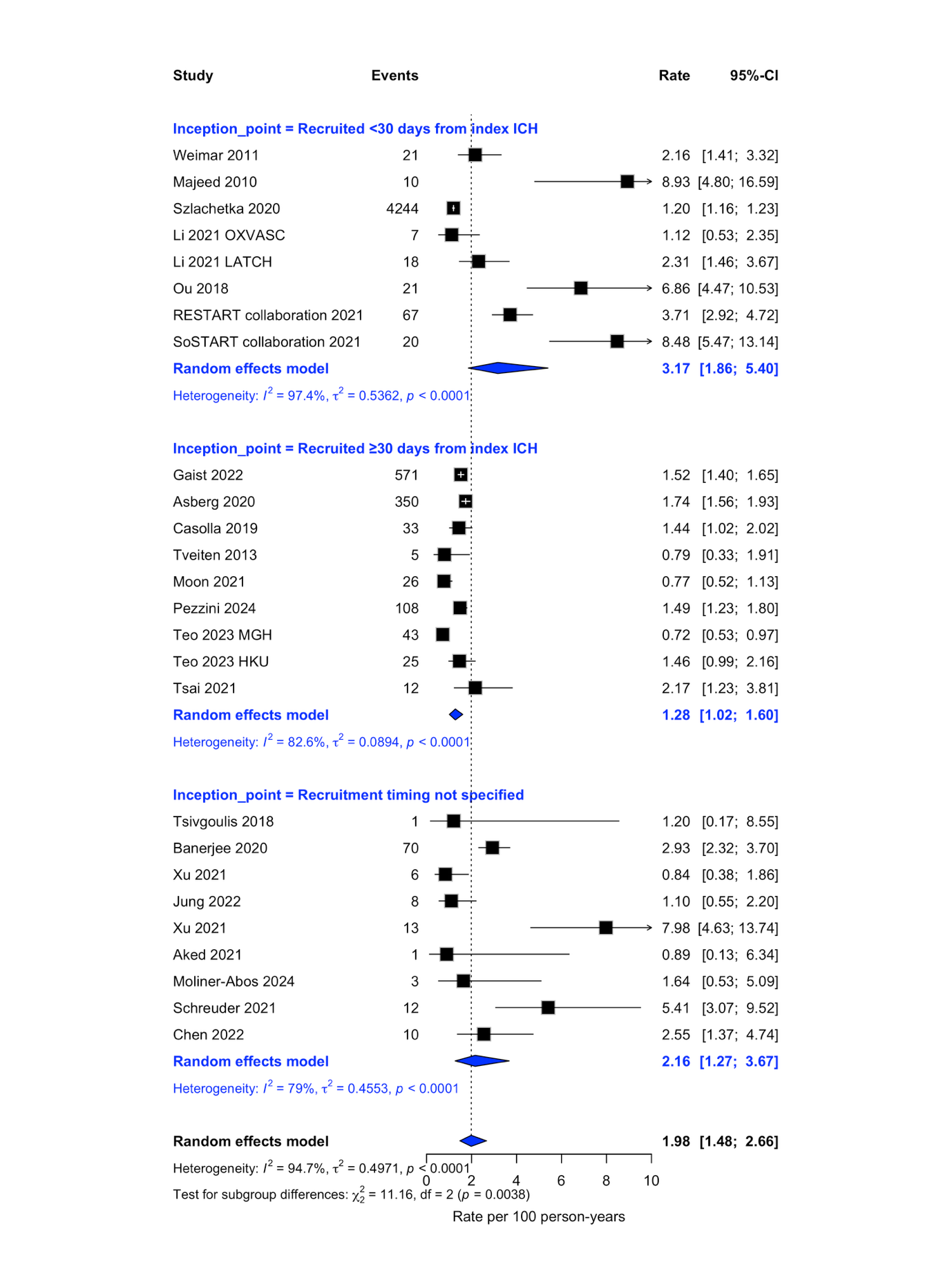


**Supplementary figure 9**: Forest plot of the annual rate of myocardial infarction sub-grouped by inception point. Studies are organized in ascending chronological order of cohort mid-year. Error bars are 95% CIs, and the diamonds represent pooled estimates.


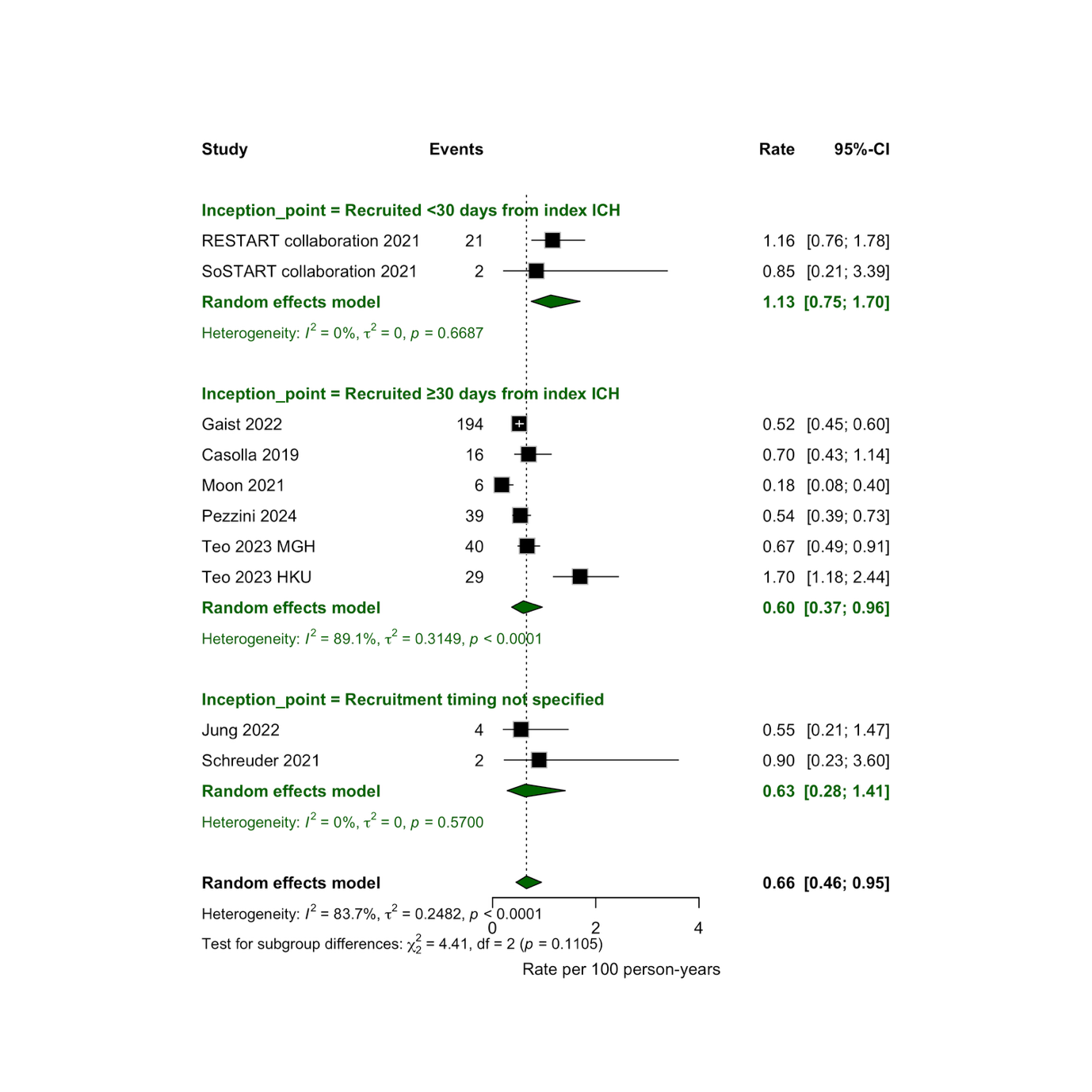


**Supplementary figure 10**: Forest plot annual rate of myocardial infarction sub-grouped by region. Studies are organized ascending chronological order of cohort mid-year. Error bars are 95% CIs, and the diamond represent the pooled estimate


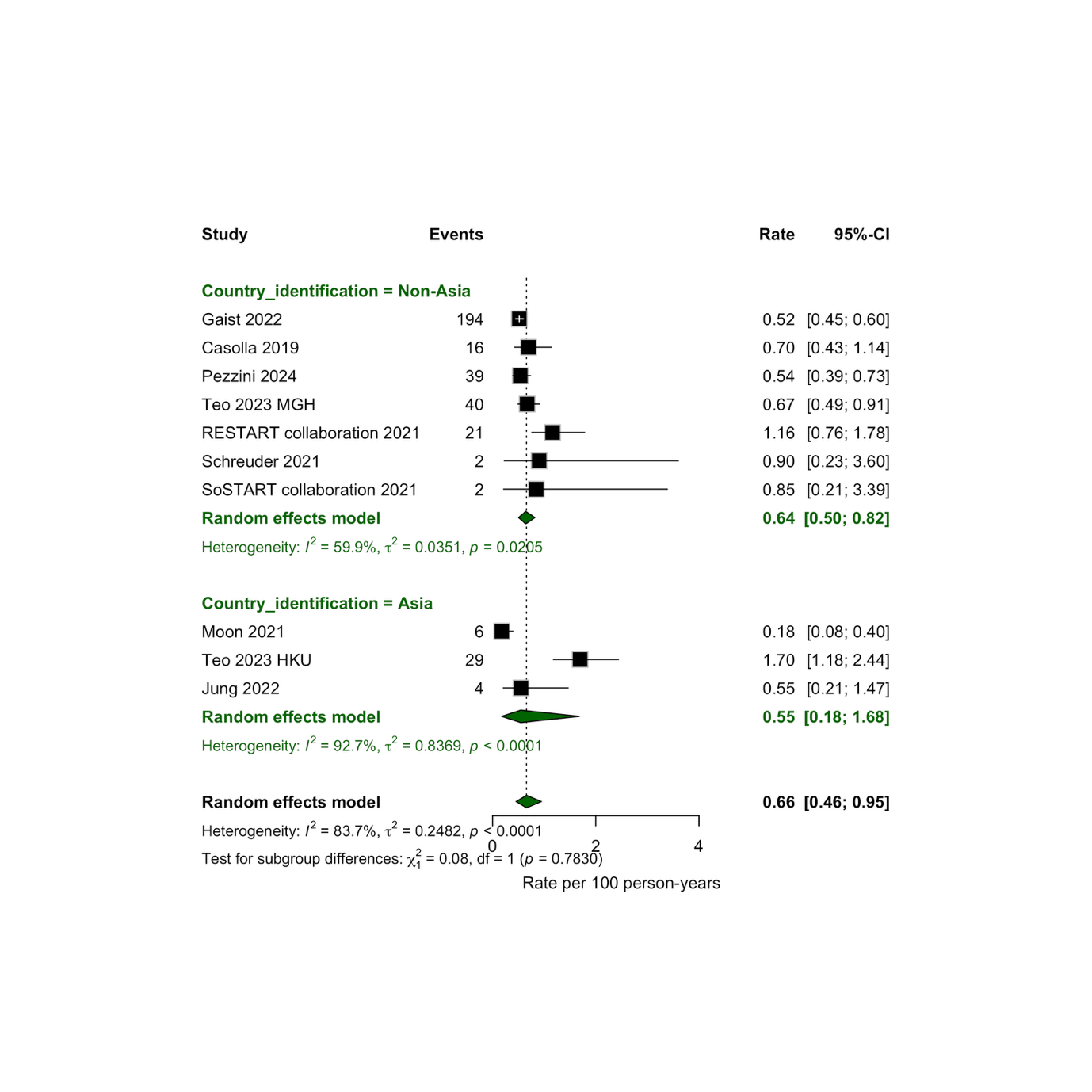


### **Supplementary figure 11:** Meta-regression analysis of the study-level estimate of outcome event rates on the study-level proportion of participants with lobar intracerebral haemorrhage


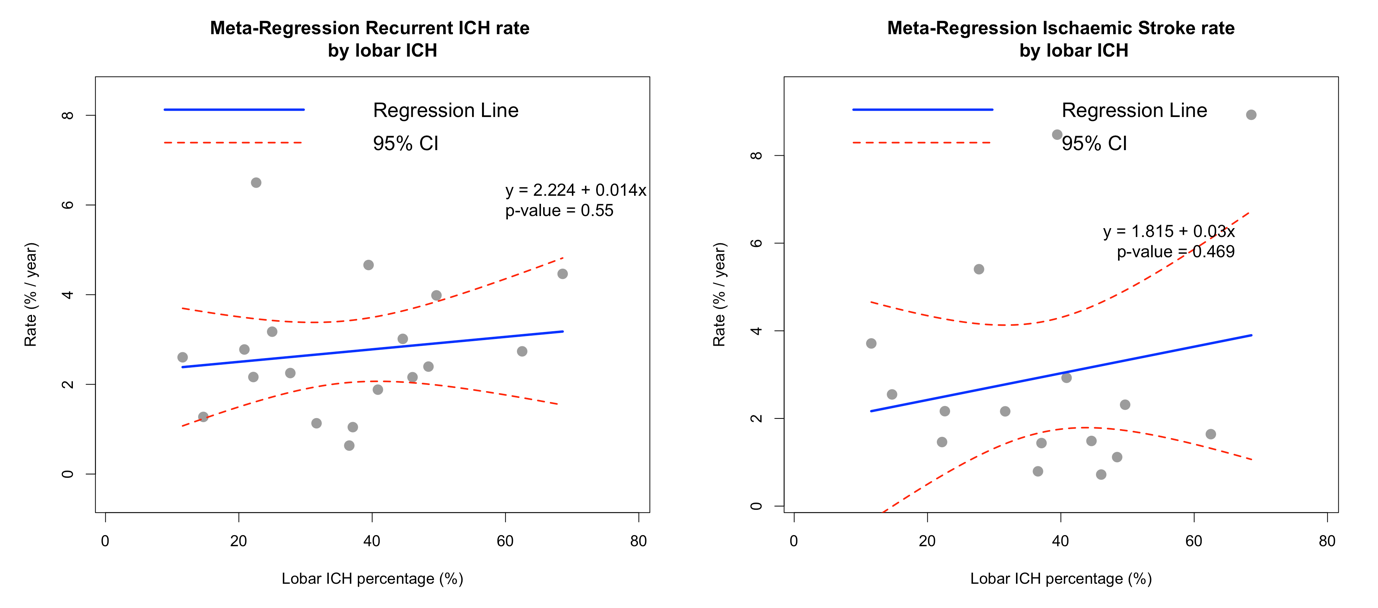


### **Supplementary figure 12**: Meta-regression analysis of the study-level estimate of outcome event rates on the study-level proportion of participant with hypertension


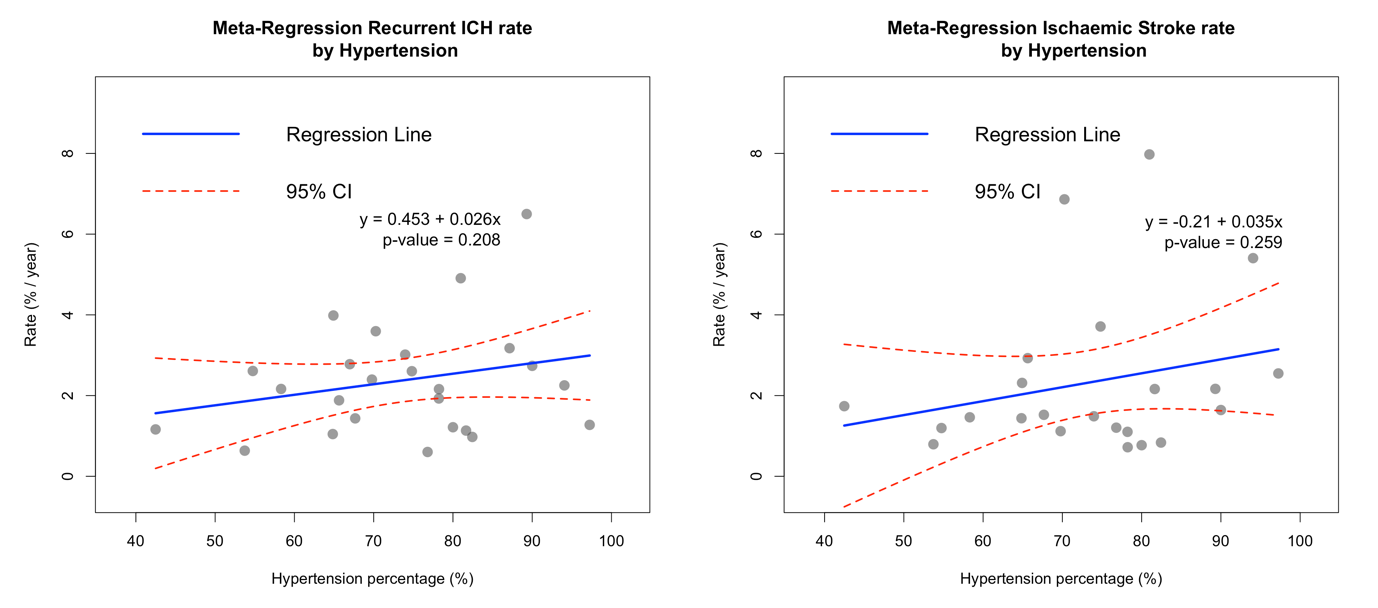


### **Supplementary figure 13**: Meta-regression analysis of the study-level estimate of outcome event rates on the study-level proportion of participant with diabetes mellitus


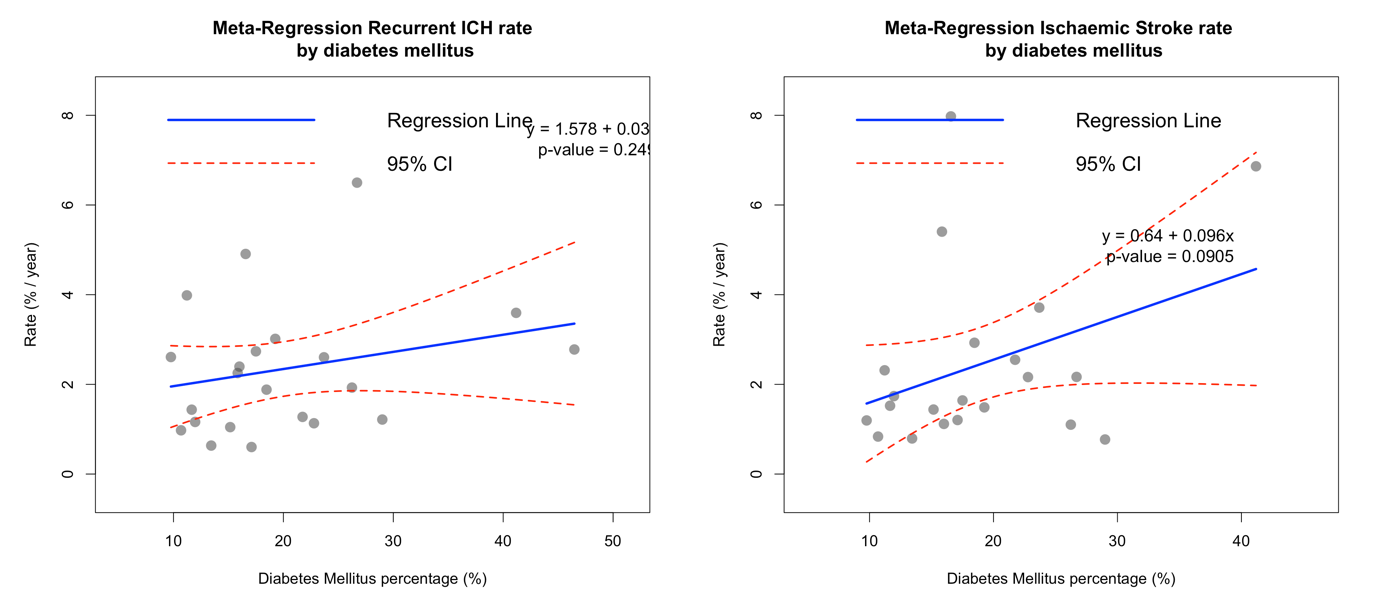


### **Supplementary figure 14**: Meta-regression analysis of the study-level estimate of outcome event rates on the study-level proportion of participant with ischaemic heart disease


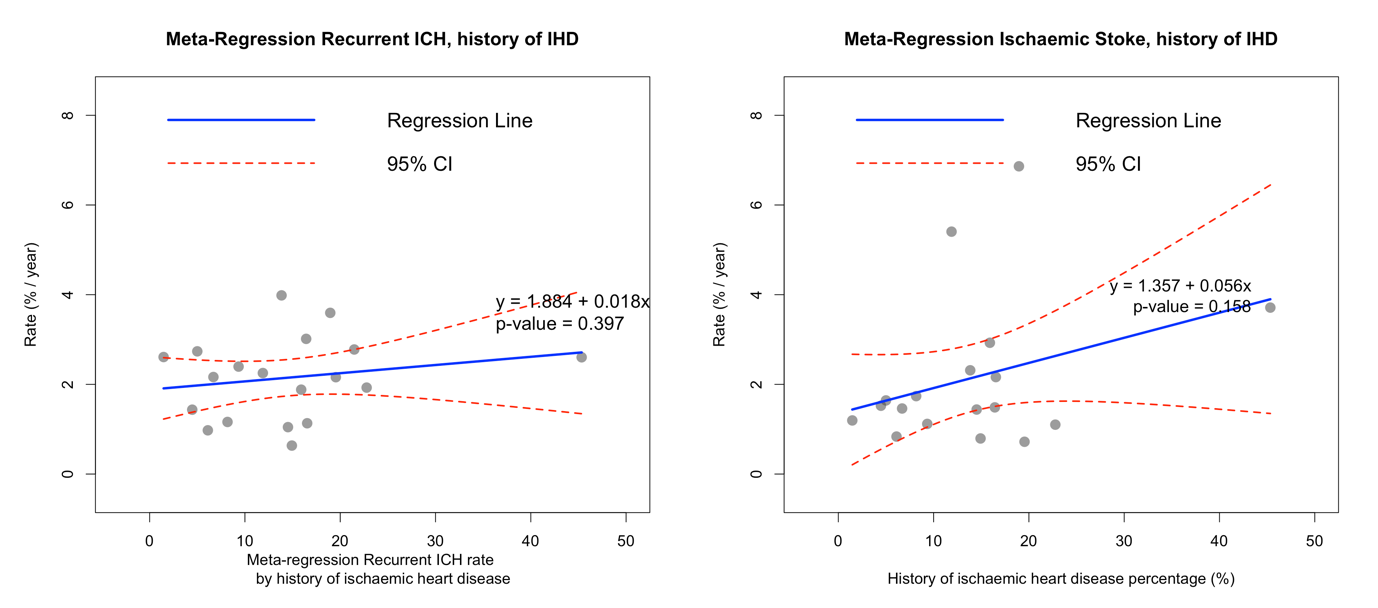


### **Supplementary figure 15:** Meta-regression analysis of the study-level estimate of outcome event rates on the study-level proportion of participant with prior ischaemic stroke or transient ischaemic attack


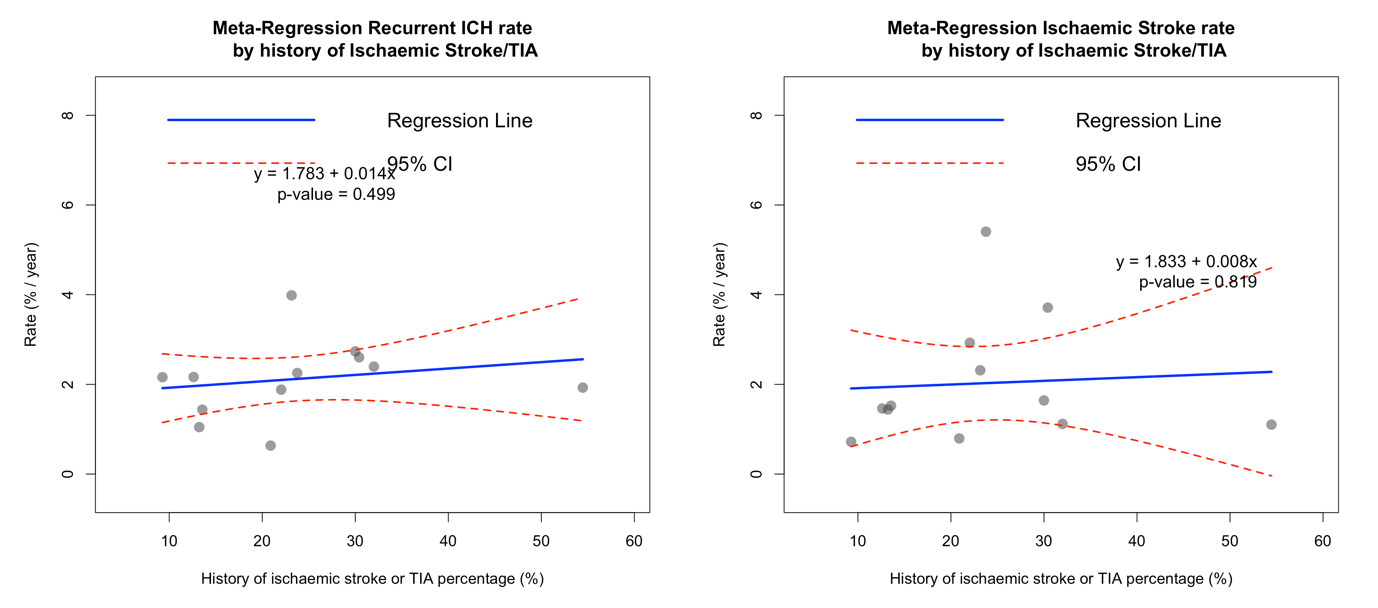


### **Supplementary figure 16:** Meta-regression analysis of the study-level estimate of outcome event rates on the study-level proportion of participant with atrial fibrillation


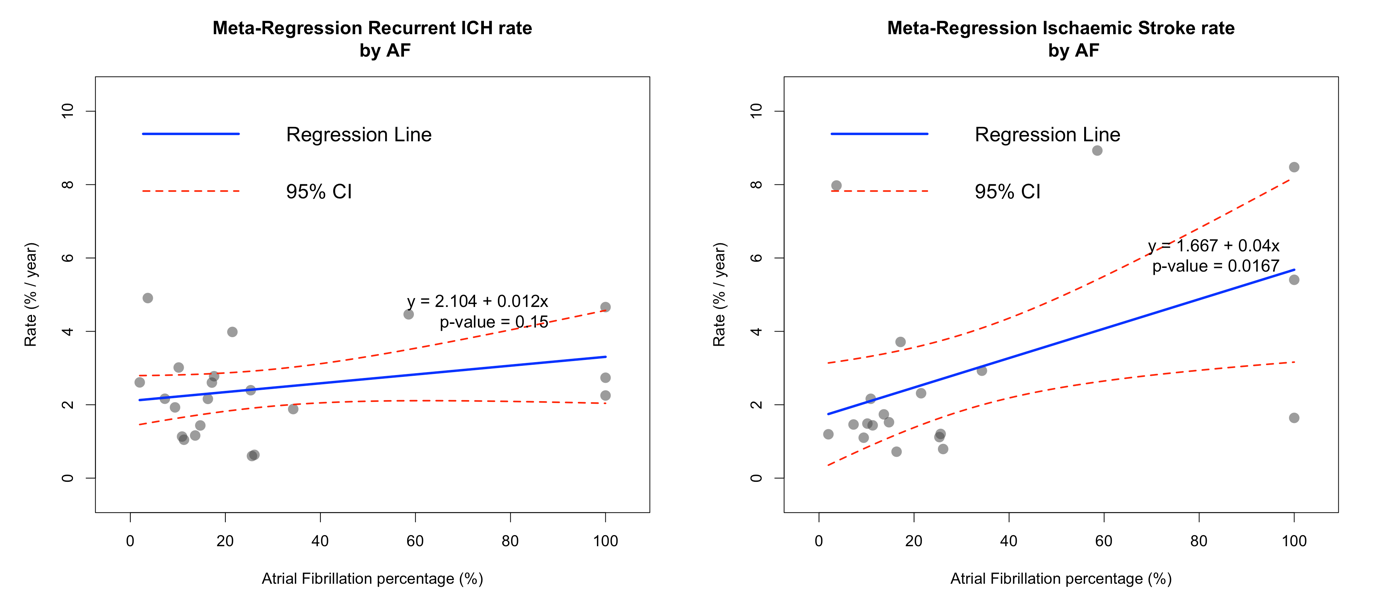


### **Supplementary table 9**: Sensitivity analysis by studies’ risk of bias and follow-up methods

|  | **Recurrent ICH** | | **Ischaemic Stroke** | |
| --- | --- | --- | --- | --- |
|  | Annual rate (95%CI) | *p* | Annual rate (95%CI) | *p* |
| **Risk of bias, overall** |  | 0.4 |  | 0.9 |
| Low | 2.41  (1.92-3.03) |  | 1.99  (1.33-2.99) |  |
| Moderate/high | 1.92  (1.39-2.66) |  | 1.98  (1.30-3.01) |  |
| **Follow up methods:** |  | 0.13 |  | 0.11 |
| Prospective | 2.26  (1.78-2.88) |  | 2.30  (1.64-3.32) |  |
| Retrospective | 1.64  (1.17-2.31) |  | 1.38  (0.82-2.33) |  |

**p*= P-values test between-group heterogeneity within each subgroup category

### **Supplementary table 10:** Sensitivity analysis by studies’ specific definition of intracerebral haemorrhage

|  | Number of cohorts | Pooled annual rate of recurrent ICH | Tau squared | Tau | I squared |
| --- | --- | --- | --- | --- | --- |
| All studies | 26 | 2.08  (1.69 - 2.56) | 0.23 | 0.48 | 94.0%  (92.3% - 95.3%) |
| After exclusion of two studies with different definitions of recurrent ICH* | 24 | 2.07  (1.68 - 2.57) | 0.22 | 0.47 | 94.1%  (92.4% - 95.4%) |

* One study considered only non-fatal recurrent ICH, and another counted intracranial hemorrhages that may include some extradural or intradural, extra parenchymal bleeding

### **Supplementary table 11**: Sensitivity analysis by studies’ specific definition of ischaemic stroke

|  | Number of cohorts | Pooled annual rate IS | Tau squared | Tau | I squared |
| --- | --- | --- | --- | --- | --- |
| Before exclusion | 26 | 1.98  (1.48 - 2.7) | 0.49 | 0.71 | 94.7%  (93.2% - 95.8% |
| After exclusion of two studies with different definitions of ischaemic stroke** | 24 | 2.03  (1.49 - 2.78) | 0.49 | 0.70 | 94.5%  (92.9% - 95.8%) |

** One study considered only non-fatal ischaemic stroke, and another counted a combination of ischaemic stroke and transient ischaemic attack

References

1. Weimar C, Benemann J, Terborg C, et al. Recurrent stroke after lobar and deep intracerebral hemorrhage: a hospital-based cohort study. *Cerebrovasc Dis* 2011; 32: 283–8.

2. Majeed A, Kim YK, Roberts RS, et al. Optimal timing of resumption of warfarin after intracranial hemorrhage. *Stroke* 2010; 41: 2860–6.

3. Casolla B, Moulin S, Kyheng M, et al. Five-Year Risk of Major Ischemic and Hemorrhagic Events After Intracerebral Hemorrhage. *Stroke* 2019; 50: 1100–1107.

4. Tveiten A, Ljostad U, Mygland A, et al. Leukoaraiosis is associated with short- and long-term mortality in patients with intracerebral hemorrhage. *J Stroke Cerebrovasc Dis* 2013; 22: 919–25.

5. Åsberg S, Farahmand B, Henriksson KM, et al. Statins as secondary preventives in patients with intracerebral hemorrhage. *Int J Stroke* 2020; 15: 61–68.

6. Gaist D, Hald SM, García Rodríguez LA, et al. Association of Prior Intracerebral Hemorrhage With Major Adverse Cardiovascular Events. *JAMA Netw Open* 2022; 5: e2234215.

7. Moon JY, Lee JG, Kim JH. Antiplatelet Therapy after Intracerebral Hemorrhage and Subsequent Clinical Events: A 12-Year South Korean Cohort Study. *Eur Neurol* 2021; 84: 183–191.

8. Pezzini A, Iacoviello L, Di Castelnuovo A, et al. Long-Term Risk of Arterial Thrombosis After Intracerebral Hemorrhage: MUCH-Italy. *Stroke* 2024; 55: 634–642.

9. Ma X, Liu D, Niu S, et al. Low-dose antiplatelet therapy survey after intracerebral hemorrhage in China: a retrospective hospital-based study. *Neurosurg Rev* 2021; 44: 2923–2931.

10. Szlachetka WA, Pana TA, Tiamkao S, et al. Impact of Diabetes on Complications, Long Term Mortality and Recurrence in 608,890 Hospitalised Patients with Stroke. *Glob Heart* 2020; 15: 2.

11. Li L, Poon MTC, Samarasekera NE, et al. Risks of recurrent stroke and all serious vascular events after spontaneous intracerebral haemorrhage: pooled analyses of two population-based studies. *Lancet Neurol* 2021; 20: 437–447.

12. Tsivgoulis G, Katsanos AH, Patousi A, et al. Stroke recurrence and mortality in northeastern Greece: the Evros Stroke Registry. *J Neurol* 2018; 265: 2379–2387.

13. Teo K-C, Keins S, Abramson JR, et al. Blood Pressure Control Targets and Risk of Cardiovascular and Cerebrovascular Events After Intracerebral Hemorrhage. *Stroke* 2023; 54: 78–86.

14. Sembolini A, Romoli M, Pannacci U, et al. Acute hematoma expansion after spontaneous intracerebral hemorrhage: risk factors and impact on long-term prognosis. *Neurol Sci* 2020; 41: 2503–2509.

15. Banerjee G, Wilson D, Ambler G, et al. Longer term stroke risk in intracerebral haemorrhage survivors. *J Neurol Neurosurg Psychiatry* 2020; 91(8): 840–845.

16. Xu M, Li B, Zhong D, et al. Cerebral Small Vessel Disease Load Predicts Functional Outcome and Stroke Recurrence After Intracerebral Hemorrhage: A Median Follow-Up of 5 Years. *Front Aging Neurosci* 2021; 13: 628271.

17. Ou R, Tang YM, Li F. The influence of enlarged perivascular spaces on the prognosis of patients with intracerebral hemorrhage. [Chinese]. *Chin J Contemp Neurol Neurosurg* 2018; 18(11): 807–812.

18. Jung NY, Cho J. Clinical effects of restarting antiplatelet therapy in patients with intracerebral hemorrhage. *Clin Neurol Neurosurg* 2022; 220: 107361.

19. Xu T, Feng Y, Wu W, et al. The Predictive Values of Different Small Vessel Disease Scores on Clinical Outcomes in Mild ICH Patients. *J Atheroscler Thromb* 2021; 28: 997–1008.

20. Al-Shahi Salman R, Dennis MS, Sandercock PAG, et al. Effects of Antiplatelet Therapy After Stroke Caused by Intracerebral Hemorrhage: Extended Follow-up of the RESTART Randomized Clinical Trial. *JAMA Neurol* 2021; 78: 1179.

21. Aked J, Delavaran H, Lindgren AG. Survival, causes of death and recurrence up to 3 years after stroke: A population-based study. *Eur J Neurol* 2021; 28: 4060–4068.

22. Tsai HH, Chen SJ, Tsai LK, et al. Long-Term Vascular Outcomes in Patients With Mixed Location Intracerebral Hemorrhage and Microbleeds. *Neurology* 2021; 96: e995–e1004.

23. Moliner-Abós C, Albertí-Vall B, Millan-Álvarez X, et al. Left atrial appendage occlusion in patients with spontaneous intracerebral hemorrhage: An observational study. *J Stroke Cerebrovasc Dis* 2024; 33: 107481.

24. Schreuder FHBM, van Nieuwenhuizen KM, Hofmeijer J, et al. Apixaban versus no anticoagulation after anticoagulation-associated intracerebral haemorrhage in patients with atrial fibrillation in the Netherlands (APACHE-AF): a randomised, open-label, phase 2 trial. *Lancet Neurol* 2021; 20: 907–916.

25. Chen CH, Chu YT, Chen YF, et al. Comparison of clinical and neuroimaging features between NOTCH3 mutations and nongenetic spontaneous intracerebral haemorrhage. *Eur J Neurol* 2022; 29: 3243–3254.

26. Al-Shahi Salman R, Keerie C, Stephen J, et al. Effects of oral anticoagulation for atrial fibrillation after spontaneous intracranial haemorrhage in the UK: a randomised, open-label, assessor-masked, pilot-phase, non-inferiority trial. *Lancet Neurol* 2021; 20: 842–853.

### Supplementary table 12: PRISMA Checklist

| **Section and Topic** | **Item #** | **Checklist item** | **Location where item is reported** |
| --- | --- | --- | --- |
| **TITLE** | | |  |
| Title | 1 | Identify the report as a systematic review. | Page 1 |
| **ABSTRACT** | | |  |
| Abstract | 2 | See the PRISMA 2020 for Abstracts checklist. | Page 3 |
| **INTRODUCTION** | | |  |
| Rationale | 3 | Describe the rationale for the review in the context of existing knowledge. | Page 4 |
| Objectives | 4 | Provide an explicit statement of the objective(s) or question(s) the review addresses. | Page 4 |
| **METHODS** | | |  |
| Eligibility criteria | 5 | Specify the inclusion and exclusion criteria for the review and how studies were grouped for the syntheses. | Page 4 |
| Information sources | 6 | Specify all databases, registers, websites, organisations, reference lists and other sources searched or consulted to identify studies. Specify the date when each source was last searched or consulted. | Page 5 |
| Search strategy | 7 | Present the full search strategies for all databases, registers and websites, including any filters and limits used. | Page 5, supplementary table 1-3 |
| Selection process | 8 | Specify the methods used to decide whether a study met the inclusion criteria of the review, including how many reviewers screened each record and each report retrieved, whether they worked independently, and if applicable, details of automation tools used in the process. | Page 5 |
| Data collection process | 9 | Specify the methods used to collect data from reports, including how many reviewers collected data from each report, whether they worked independently, any processes for obtaining or confirming data from study investigators, and if applicable, details of automation tools used in the process. | Page 5 |
| Data items | 10a | List and define all outcomes for which data were sought. Specify whether all results that were compatible with each outcome domain in each study were sought (e.g. for all measures, time points, analyses), and if not, the methods used to decide which results to collect. | Page 5 |
|  | 10b | List and define all other variables for which data were sought (e.g. participant and intervention characteristics, funding sources). Describe any assumptions made about any missing or unclear information. | Page 5-6 |
| Study risk of bias assessment | 11 | Specify the methods used to assess risk of bias in the included studies, including details of the tool(s) used, how many reviewers assessed each study and whether they worked independently, and if applicable, details of automation tools used in the process. | Page 6 |
| Effect measures | 12 | Specify for each outcome the effect measure(s) (e.g. risk ratio, mean difference) used in the synthesis or presentation of results. | Page 5-6 |
| Synthesis methods | 13a | Describe the processes used to decide which studies were eligible for each synthesis (e.g. tabulating the study intervention characteristics and comparing against the planned groups for each synthesis (item #5)). | Page 5-6 |
|  | 13b | Describe any methods required to prepare the data for presentation or synthesis, such as handling of missing summary statistics, or data conversions. | Page 5-6 |
|  | 13c | Describe any methods used to tabulate or visually display results of individual studies and syntheses. | Page 5-6 |
|  | 13d | Describe any methods used to synthesize results and provide a rationale for the choice(s). If meta-analysis was performed, describe the model(s), method(s) to identify the presence and extent of statistical heterogeneity, and software package(s) used. | Page 5-6 |
|  | 13e | Describe any methods used to explore possible causes of heterogeneity among study results (e.g. subgroup analysis, meta-regression). | Page 5-6 |
|  | 13f | Describe any sensitivity analyses conducted to assess robustness of the synthesized results. | Page 6 |
| Reporting bias assessment | 14 | Describe any methods used to assess risk of bias due to missing results in a synthesis (arising from reporting biases). | - |
| Certainty assessment | 15 | Describe any methods used to assess certainty (or confidence) in the body of evidence for an outcome. | - |
| **RESULTS** | | |  |
| Study selection | 16a | Describe the results of the search and selection process, from the number of records identified in the search to the number of studies included in the review, ideally using a flow diagram. | Page 6-7, supplementary figure 1 |
|  | 16b | Cite studies that might appear to meet the inclusion criteria, but which were excluded, and explain why they were excluded. | Page 6 |
| Study characteristics | 17 | Cite each included study and present its characteristics. | Page 6, 10, supplementary table 4 |
| Risk of bias in studies | 18 | Present assessments of risk of bias for each included study. | Supplementary figure 2-3 |
| Results of individual studies | 19 | For all outcomes, present, for each study: (a) summary statistics for each group (where appropriate) and (b) an effect estimate and its precision (e.g. confidence/credible interval), ideally using structured tables or plots. | Page 10-12 |
| Results of syntheses | 20a | For each synthesis, briefly summarise the characteristics and risk of bias among contributing studies. | Page 5 |
|  | 20b | Present results of all statistical syntheses conducted. If meta-analysis was done, present for each the summary estimate and its precision (e.g. confidence/credible interval) and measures of statistical heterogeneity. If comparing groups, describe the direction of the effect. | Page 11-12, supplementary table 8 |
|  | 20c | Present results of all investigations of possible causes of heterogeneity among study results. | Page 6-7, 11,12, Supplementary table 8, |
|  | 20d | Present results of all sensitivity analyses conducted to assess the robustness of the synthesized results. | Page 7, Supplementary table 9- 11 |
| Reporting biases | 21 | Present assessments of risk of bias due to missing results (arising from reporting biases) for each synthesis assessed. | Supplementary table 9 |
| Certainty of evidence | 22 | Present assessments of certainty (or confidence) in the body of evidence for each outcome assessed. | - |
| **DISCUSSION** | | |  |
| Discussion | 23a | Provide a general interpretation of the results in the context of other evidence. | Page 7 |
|  | 23b | Discuss any limitations of the evidence included in the review. | Page 8 |
|  | 23c | Discuss any limitations of the review processes used. | Page 8 |
|  | 23d | Discuss implications of the results for practice, policy, and future research. | Page 8-9 |
| **OTHER INFORMATION** | | |  |
| Registration and protocol | 24a | Provide registration information for the review, including register name and registration number, or state that the review was not registered. | Page 5 |
|  | 24b | Indicate where the review protocol can be accessed, or state that a protocol was not prepared. | Page 5 |
|  | 24c | Describe and explain any amendments to information provided at registration or in the protocol. | N/A |
| Support | 25 | Describe sources of financial or non-financial support for the review, and the role of the funders or sponsors in the review. | 2 |
| Competing interests | 26 | Declare any competing interests of review authors. | 2 |
| Availability of data, code and other materials | 27 | Report which of the following are publicly available and where they can be found template data collection forms; data extracted from included studies; data used for all analyses; analytic code; any other materials used in the review. | - |
